# R5 HIV-1 envelope attracts dendritic cells to cross the human intestinal epithelium and sample luminal virions via engagement of the CCR5

**DOI:** 10.1002/emmm.201202232

**Published:** 2013-04-22

**Authors:** Mariangela Cavarelli, Chiara Foglieni, Maria Rescigno, Gabriella Scarlatti

**Affiliations:** 1 Unit of Viral Evolution and Transmission, DITID, San Raffaele Scientific InstituteMilan, Italy; 2 Department of Experimental Oncology, European Institute of OncologyMilan, Italy

**Keywords:** dendritic cells, HIV-1, mucosal transmission

## Abstract

The gastrointestinal tract is a principal route of entry and site of persistence of human immunodeficiency virus type 1 (HIV-1). The intestinal mucosa, being rich of cells that are the main target of the virus, represents a primary site of viral replication and CD4^+^ T-cell depletion. Here, we show both *in vitro* and *ex vivo* that HIV-1 of R5 but not X4 phenotype is capable of selectively triggering dendritic cells (DCs) to migrate within 30 min between intestinal epithelial cells to sample virions and transfer infection to target cells. The engagement of the chemokine receptor 5 on DCs and the viral envelope, regardless of the genetic subtype, drive DC migration. Viruses penetrating through transient opening of the tight junctions likely create a paracellular gradient to attract DCs. The formation of junctions with epithelial cells may initiate a haptotactic process of DCs and at the same time favour cell-to-cell viral transmission. Our findings indicate that HIV-1 translocation across the intestinal mucosa occurs through the selective engagement of DCs by R5 viruses, and may guide the design of new prevention strategies.

## INTRODUCTION

The mucosal surface of the gastrointestinal tract is the main route of entry of human immunodeficiency virus type 1 (HIV-1) during mother-to-child transmission (MTCT) via ingestion of infected biological fluids as well as during anal sexual intercourses. Regardless of the route of transmission, the intestinal tissue, being a major site of viral replication, is rapidly affected by a conspicuous CD4^+^ T-cell depletion after primary infection (Mattapallil et al, 2005; Schneider et al, 1995; Wang et al, 2007). CD4^+^ T cells fail to fully repopulate the gastrointestinal tract despite an effective antiretroviral therapy (ART) (Chun et al, 2008), and the virus is detected in the small and large intestines although absent from blood circulation (Ling et al, 2010). Microbial translocation, revealed by circulating LPS during chronic HIV infection, testifies the continuous damage of this tissue (Brenchley et al, 2006; Redd et al, 2009).

The possible suggested pathways for HIV-1 to cross the intestinal epithelial monolayer, which could eventually bias the transmission of certain viral variants, would be: the occurrence of breaches in the epithelium, which interrupt the continuity of the tissue; transcytosis through epithelial cells; viral uptake by specialized epithelial cells such as M cells or by direct sampling by dendritic cells (DCs) of the lamina propria. I*n vitro* studies support some of these mechanisms. Cell-free and cell-associated viruses of R5 or X4 phenotype are taken up via binding to the galactosyl ceramide (GalCer) receptor and transcytosed by colonic epithelial cells (Bomsel, 1997). However, primary jejunal epithelial cells incubated with HIV-1 carry over only R5 viruses to receptive target cells (Meng et al, 2002), whereas M cells transport selectively X4 viral variants through a chemokine-receptor mediated mechanism (Fotopoulos et al, 2002). In addition, DCs in jejunum explant cultures are the predominant target cell of R5 HIV-1 early after infection, and leave the tissue to transmit in *trans* the virus to lymphocytes (Shen et al, 2010). Thus, some of the described mechanisms support a preferential transmission of CCR5-using viruses, which reflect the *in vivo* prevalence of R5 variants during the acute infection (Cavarelli et al, 2008; Koot et al, 1993; Scarlatti et al, 1997), others instead provided evidence of the transmission of X4 viruses as well.

In non-human primate (NHP) studies, the infection of the genital epithelium pointed to DCs as first target cells for the virus (Hu et al, 2000; Spira et al, 1996). Infected DCs were detected in the pluristratified cervico-vaginal epithelium within 60 min from viral exposure, and thereafter accumulated within 2–3 days beneath the epithelium (Hu et al, 2000; Spira et al, 1996). In a recent study, the expression of the chemokine CCL20 in the endocervical epithelium after viral exposure suggested its involvement as an outside-in signal for the sub-epithelial recruitment of plasmacytoid DCs (pDCs) and CD4^+^ T cells (Li et al, 2009). On the other hand, studies performed in mice with microbes other than HIV demonstrated that the release of fractalkine by intestinal epithelial cells induced DCs to extend cellular projections across the intact intestinal epithelium and translocate bacteria to the lamina propria (Niess et al, 2005; Rescigno et al, 2001). Taken together, these studies suggest that multiple factors may be involved in early HIV-1 infection.

Here, we address the question of how DCs are involved in HIV-1 infection at intestinal mucosal level. We show that DCs have an active role in the infection mechanism of the mucosal tissue, as they are selectively recruited by R5 HIV-1 through the mucosa and then act as reservoir of viruses. We propose a model in which HIV-1 can transiently open tight junctions (TJs) between epithelial cells to generate a viral gradient that drives migration of DCs via CCR5. The close contact between DCs and epithelial cells may also favour cell-to-cell viral spread.

## RESULTS

### R5 HIV-1 induce migration of DCs through a tight monolayer of intestinal epithelial cells

**Figure 1:**
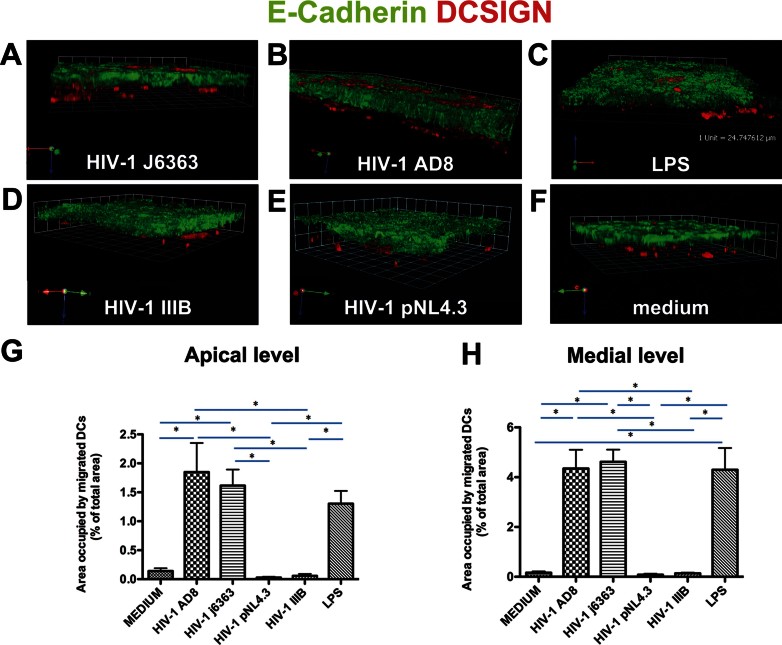
R5 but not X4 HIV-1 induces DCs to migrate through a monolayer of epithelial cells. Caco-2 cells were grown on transwell filter to form a confluent monolayer then DCs were let to adhere to the bottom of the filter. Cell-free HIV-1, LPS (1 µg/ml) or medium (DMEM 10% FCS) were incubated on the apical side of the Caco-2 monolayer for 1.5 h. Filters were processed for CM. A–F. Three-dimensional rendering of representative fields obtained with Volocity 5.0 software. Mouse anti-human E-Cadherin + Alexafluor488 goat anti-mouse IgG2a (green) depicts interepithelial adherent junctions. Mouse anti-human DC-SIGN-PE (red) labels the DCs, which creep through epithelial cells in response to incubation with R5 HIV-1^J6363^ (A, at 1 ng of p24 Ag) and HIV-1^AD8^ (B, at 24 ng of p24 Ag) but do not in response to X4 HIV-1^IIIB^ (D, at 10 ng of p24 Ag) and HIV-1^pNL4.3^ (E, at 40 ng of p24 Ag). LPS (C) and medium (F) were positive and negative controls. The experiment was repeated three times. G,H. Quantitative analysis of DCs migration across the Caco-2 cell monolayer at the apical (G) and medial (H) level of the cell layer is shown. Results are expressed as percentage of area occupied by DCs compared to that of the whole field. Bars represent mean ± SD of three or four fields of three different experiments. Statistic analysis was performed as described in Materials and Methods Section. *p < 0.05.

To test the hypothesis that HIV-1 can gain access into the intestinal mucosa by inducing DCs to send cellular projections across the epithelial cell monolayer and sample luminal virions, we developed a dual-chamber Caco-2/DCs *in vitro* co-culture system. Cell-free HIV-1 of R5 but not of X4 phenotype, when added to the apical surface of the intestinal epithelial Caco-2 cell culture, induced an intense migration of DCs across the monolayer to a level comparable to or higher than the positive control LPS as shown with confocal microscopy (CM) (Fig 1). This phenomenon was reproduced with three R5 viruses (subtype B), the primary isolate HIV-1^J6363^ (Fig 1A) and the pseudoviruses HIV-1^AD8^ and HIV-1^YU2^ (Fig 1B and Fig S1 of Supporting Information) but was not induced with three X4 viruses (2 subtype B and one D), the isolate HIV-1^IIIB^ (Fig 1D) and the pseudoviruses HIV-1^pNL4.3^ and HIV-1^92UG024^ (Fig 1E and Fig S1 of Supporting Information). Virus input as low as 1 ng of p24 antigen (Ag) was enough to engage DCs. No migration or some sporadic spontaneous elongation of DCs was observed with the negative control medium (Fig 1F), as well as mock cultures of PBMCs and mock transfected 293T cells (data not shown).

To determine the amount of DCs that migrated across the epithelium, we calculated the area occupied by DCs at the apical (Fig 1G) and medial (Fig 1H) level of the Caco-2 cells monolayer. The amount of DC migration induced by the R5 viruses HIV-1^AD8^ and HIV-1^J6363^ was significantly different compared to the X4 HIV-1^pNL4.3^, HIV-1^IIIB^ and medium (*p* < 0.05) but not to the positive control LPS. The repeated measurements (*n* = 3 or 4) of each treatment were homogenous (*p* = 0.537, at apical level and *p* = 0.918, at medial level) thus validating the technical approach.

In our experimental system, DCs have minimal expression of CXCR4, which may limit the DC migration in response to X4 viruses. To exclude that the non-responsiveness of the DCs to X4 viruses was merely due to low level of CXCR4 expression, DCs were induced to express comparable levels of CCR5 and CXCR4, than their migration across the epithelial monolayer in response to HIV stimulation tested. The experiments confirmed our previous results that DC migration was induced exclusively by R5 and not X4 HIV-1 (Fig S2 of Supporting Information).

Time course experiments revealed that the kinetic of dendrites elongation and DC migration induced by R5 viruses was fast: the effect was visible already at 30 min and conspicuously increased over a time course of 4.5 h (Fig S3 of Supporting Information). Complete transmigration with DCs on the top of the epithelial monolayer was most evident with long incubation times, but migrated DCs were not massively released into the apical chamber. Indeed, after 4 h of incubation with HIV-1^AD8^ only a limited number of DCs (4 ± 5 cells) were detected in the apical chamber compared to none in the negative control with medium. DCs expressed the TJ proteins Occludin and ZO-1, the Junction-Adhesion-Molecule (JAM) and the β2 integrin LFA-1 (Fig S4 of Supporting Information), which may favour retainment of DCs attached to the epithelium.

**Figure 2:**
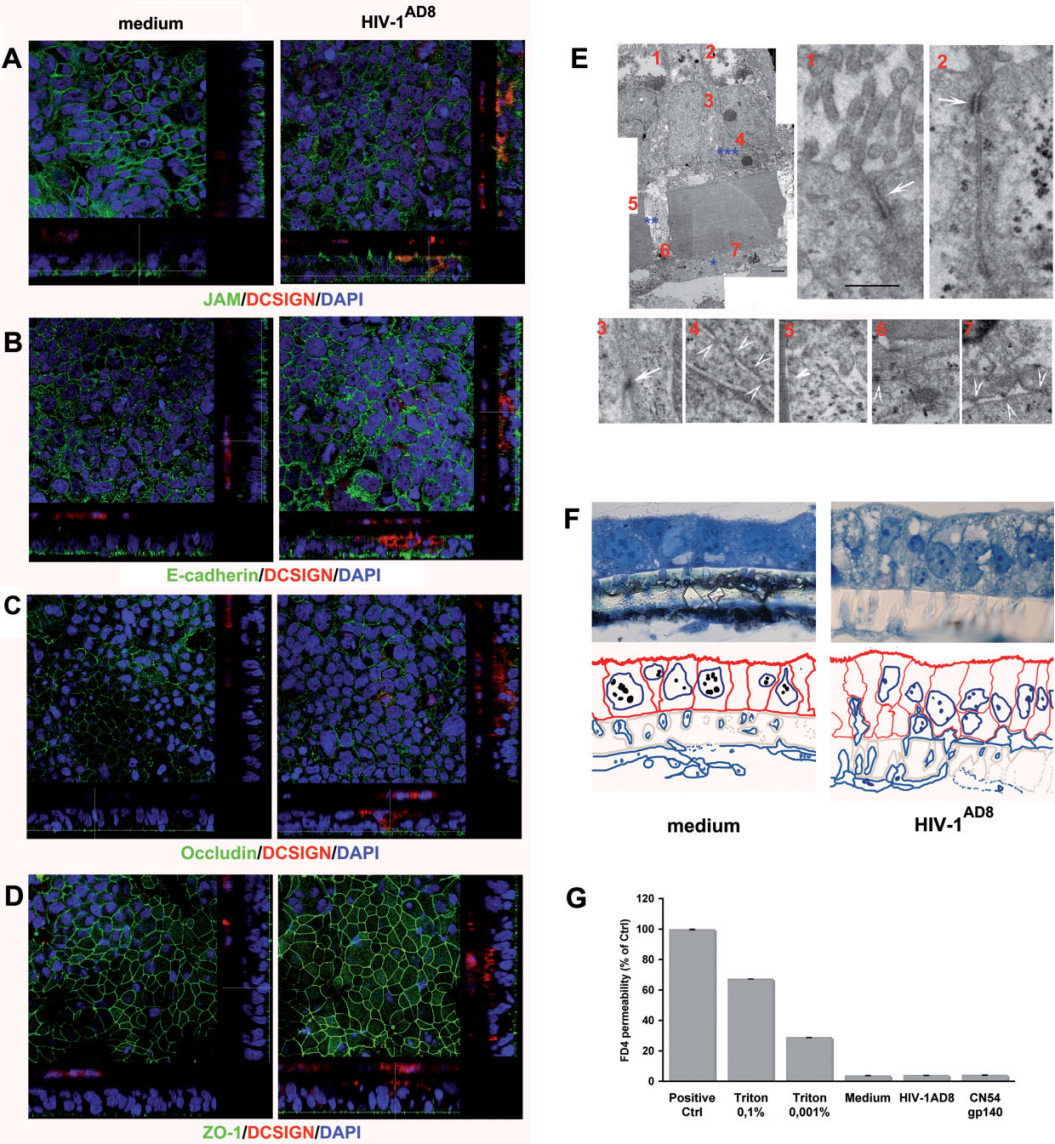
DCs migration does not alter junctional protein expression by Caco-2 cells and preserves the integrity of the HIV-1 treated monolayer. The Caco-2/DCs co-culture system was incubated with medium or R5 HIV-1^AD8^ at 24 ng of p24 Ag for 1.5 h. A–D. CM cross-sectional images of specimens stained for DCSIGN-PE (red) and for the epithelial junctions (green) JAM (A), E-Cadherin (B) Occludin (C) ZO-1 (D) showed that HIV-1 as well as migrated DCs did not affect the intraepithelial junctions expression in the Caco-2/DC system. DAPI stained the nuclei. Results are from one representative experiment out of three. E. Ultrastructure of Caco-2/DCs culture treated with R5 HIV-1^J6363^ (at 1 ng of p24 Ag) for 1.5 h. TEM images show DCs adhering to the filter (*), DCs protrusions inside the filter membrane pore (**) and a DC interposed between adjacent Caco-2 cells (***) Scale bar: 2 µm. Numbers identify the corresponding magnified images displayed in panel (E), and arrows point to interepithelial TJs (1, 2), TJs-like structures between DCs and Caco-2 cells (3–5), contiguity among cells inside the pore (6), and junction like-structures between DCs (7) Scale bar: 500 nm. Results from a representative experiment out of three are shown. F. Semi-thin sections for TEM labelled with Toluidine blue and the corresponding explicative colour mask below (Caco-2 cells red, DCs blue, filter grey) show the morphology and the spatial organization of cells. Caco-2 cells are columnar and polarized, displaying microvilli, dense cytoplasmic granules and vacuoles characteristic of epithelial cells. DCs are disposed along the lower face of the filter, inside the membrane pores and, in HIV-1 treated sample, intercalated in between Caco-2 cells, without destroying epithelial monolayer continuity. G. The permeability of the Caco-2/DCs culture to FD4 (4 kDa, 250 µg/ml) in the presence of medium, R5 HIV-1^AD8^ (at 24 ng of p24 Ag) and CN54 gp140 protein (100 ng/ml) is shown as percentage of the positive control (*i.e.* FD4 added in the upper chamber of the transwell without Caco-2 cells). Triton X100 was included as control of barrier disruption. Results are mean values ± SD of triplicates from a representative experiment out of three.

At the conditions generally used in the experiments, *i.e.* 1.5 h of virus incubation, the epithelium maintained a regular expression of Occludin, ZO-1, JAM and E-Cadherin (Fig 2A–D). Moreover the integrity of the monolayer and the organization of the epithelial cell brush-border was preserved as in the control, and TJ-like structures between DCs and Caco-2 cells were observed (Fig 2E and F and Fig S5 of Supporting Information). This is in line with our previous results in mice that DCs migrating between epithelial cells following stimulation with bacteria preserve the epithelial barrier due to TJ-like structures between DCs and Caco-2 cells (Rescigno et al, 2001). To further substantiate tightness between cells, we determined the permeability to FITC-dextran (FD4). The amount of translocated FD4 was comparable at 1.5 h in all conditions used (mean 3.82 ± 0.18, 3.98 ± 0.19 and 3.67 ± 0.23% in the culture viral supernatant-, gp140 protein- and medium-treated cultures, respectively) (Fig 2G). Of note, an overnight incubation disrupted the monolayer not only of the treated cultures but also of the negative controls, which supports the importance to perform time-controlled experiments.

**Figure 3:**
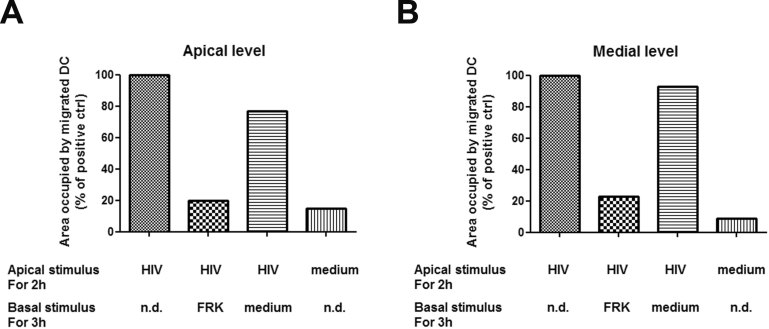
DCs migration across a Caco-2 monolayer is reversible. Caco-2 cells were grown on transwell filter to form a confluent monolayer then DCs were let to adhere to the bottom of the filter. Cell-free R5 HIV-1^AD8^ or medium were incubated on the apical side of the Caco-2 monolayer for 2 h (Apical stimulus). After extensive wash, samples were either immediately fixed with 2% PFA, or the transwell moved in a new plate containing either medium or fractalkine (FRK, 100 ng/ml) in the basal chamber and further incubated for 3 h before fixation (Basal stimulus). Quantitative analysis of DCs migration across the Caco-2 cell monolayer at the apical (A) and medial (B) level of the cell layer is shown. Results are the mean of three replicates each and are expressed as percentage of the positive control (*i.e.* area occupied by DCs following apical treatment with HIV-1). n.d., not done.

We sought to investigate whether DCs, once migrated into the epithelial monolayer, were able to return to the basal chamber after an appropriate stimulus, such as the CX3CR1-binding chemokine fractalkine, known to induce DCs migration (Dichmann et al, 2001). As shown in Fig 3, incubation with fractalkine completely reversed the migration of DCs to the basal level. Thus, DCs can shuttle across the epithelial barrier and thus vehicle captured virus to replication competent cells in the mucosa or lymph nodes.

### *Ex vivo* as *in vitro*: R5 viruses recruit selectively DCs

**Figure 4:**
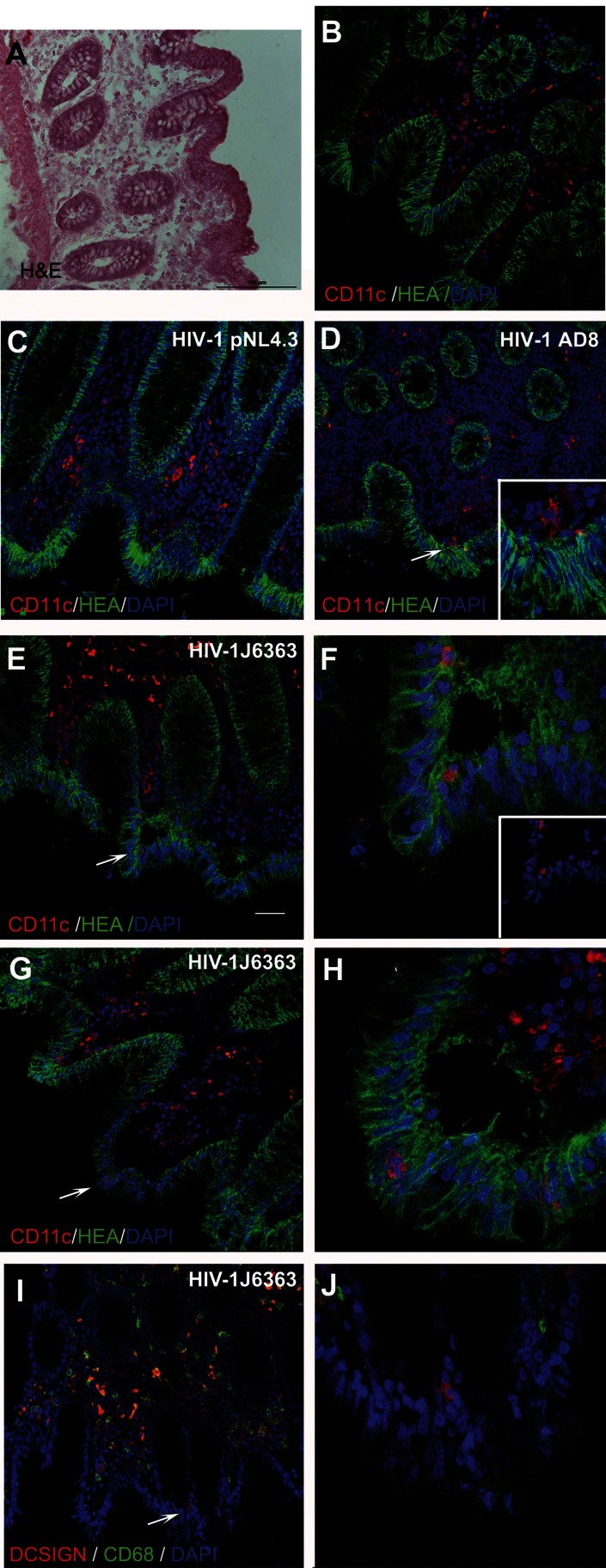
R5 HIV-1 induces migration of DCs through the colonic epithelium. Colonic tissue was either left untreated (A and B) or incubated with X4 HIV-1^pNL4.3^ (C), R5 HIV-1^AD8^ (D) or R5 HIV-1^J6363^ (E–J) (at 50 ng of p24 Ag) for 30 min. CD11c^+^ cells were detected in the colonic lamina propria of untreated (B) and HIV-1^pNL4.3^ treated (C) tissues but not in between epithelial cells. Following R5 HIV-1 incubation protruded dendrites (D) or whole DCs (E–H) were observed inside the epithelium. Moreover DCs (DCSIGN^+^/CD68^−^ cells) but not Mϕ (DCSIGN^+^/CD68^+^ and DCSIGN^-^CD68^+^ cells) migrated through the epithelium upon HIV-1 stimulation (I and J). Cryosections were fixed with 4% PFA and immunostained with Hematoxylin–eosin (A), or for human epithelial antigen (mouse anti-HEA-FITC, green), DAPI (nuclei; blue) and either mouse anti-human CD11c + Alexafluor594 goat anti-mouse IgG (DCs; red) (B–H) or mouse anti-human DC-SIGN-PE (DCs and Mϕ; red) and mouse anti-human CD68^+^ Alexafluor488 goat anti-mouse IgG1k (monocytes/Mϕ; green) (I and J). The inset in panel (D) and (F) is magnified 3×. Panels (F), (H) and (J) are magnification (zoom 3×) of the area indicated by arrow in (E), (G) and (I), respectively. The inset of panel (F) evidences DCs after hiding of HEA channel. Scale bar: 50 µm in B–E, G and I. Each Figure is representative of results from five donor tissues.

To assess the relevance of our observations beyond an *in vitro* culture model, we developed an *ex vivo* polarized human colonic tissue culture. Also in this system, only R5 viruses (HIV-1^AD8^ and HIV-1^J6363^) and not X4 viruses (HIV-1^pNL4.3^ and HIV-1^IIIB^), when incubated on the apical side of the sealed organ culture, induced migration of lamina propria resident DCs to the villi's apical edges within 30 min (Fig 4 and data not shown). DCs migrated close to the basal membrane of the epithelium extending cellular protrusions (Fig 4D) or within the epithelial cells (Fig 4E–I). The intraepithelial migration of DCs was rapid but transient as no DCs were detected at longer times of viral incubation (*i.e.* 1, 3 or 5 h). DCs were identified either as CD11c^+^ (Fig 4C–H) or DCSIGN^+^CD68^−^ cells (Fig 4I and J) to distinguish them from Mϕ. In contrast to DCs, lamina propria resident Mϕ (DCSIGN^+^CD68^+^ and DCSIGN^-^CD68^+^) did not creep between epithelial cells even at incubation times as long as 5 h (Fig 4I and J), providing evidence of the unique role of DCs in this process. Thus, we confirmed with the *ex vivo* tissue culture the results obtained with the *in vitro* model: lamina propria DCs were capable to creep in the colonic epithelium in response to HIV-1 R5 virus but not X4 virus.

### HIV-1 is captured by DCs migrating across the intestinal epithelium

**Figure 5:**
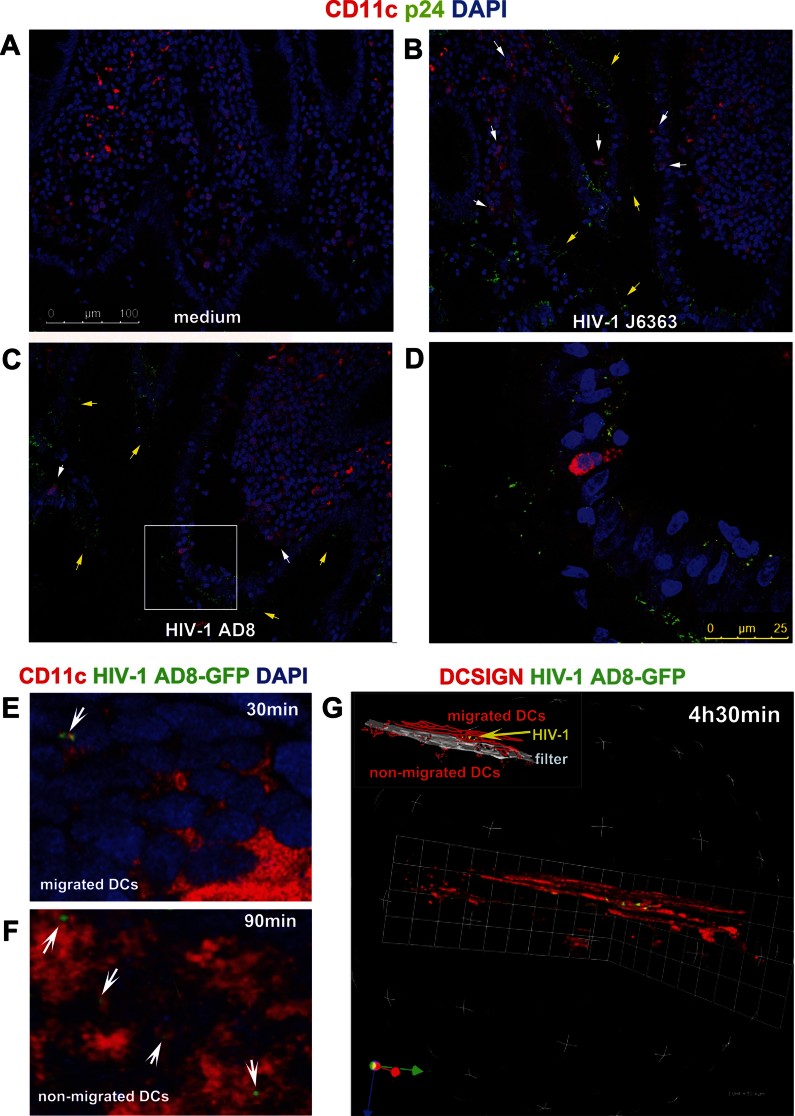
DCs capture HIV-1 in colonic explant and *in vitro* Caco-2/DC model. Human colonic tissue was either left untreated (A) or incubated with R5 HIV-1^J6363^ (B) or R5 HIV-1^AD8^ (C and D) (at 50 ng of p24 Ag) for 30 min. HIV-1 virions co-localized with CD11c^+^ DCs that migrate inside the epithelium as well as with DCs closely underlying the epithelium (see white arrows in B and C). Moreover virions were detected at both the apical and basal side as well as penetrating the epithelium. Yellow arrows point to virions entrapped in the mucus. Absence of p24 Ag in the basal medium, determined with ELISA, confirmed the seal integrity of the tissue culture system without any viral leakage (as described in Materials and Methods Section). Panel (D) is a magnification (zoom 3×) of the boxed area in C. Cryosections were fixed with 4% PFA and stained with mouse anti-human CD11c-PE (DCs; red), mouse anti-p24 + Alexafluor488 goat anti-mouse IgG (HIV-1; green), and DAPI (nuclei; blue). Scale bar indicate the magnifications. Each Figure is representative of results from three donor tissues. Caco-2/DCs co-culture was incubated with R5 HIV-1^AD8-GFP^ (at 20 ng of p24 Ag) for 30 min (E), 1.5 h (F) or 4.5 h (G). (A and B) Three-dimensional renderings from CM *z*-series stacks of representative images (acquired with objective 40×, zoom 6.5×) showed GFP-expressing virions (green, indicated by arrows) either on migrated (E) as well as non-migrated DCs at the basal side (F, the bottom side of the filter is shown). Cells were stained for DAPI (all cells; blue) and mouse anti-human CD11c + AlexaFluor594 goat anti-mouse IgG (DCs; red). (G) Three-dimensional rendering from a CM *z*-series stack of images showed a cluster of DCs (visualized with mouse anti-human DC-SIGN-PE; red) migrated to the Caco-2 side of the culture (Caco-2 cells not shown in the Figure) after incubation with HIV-1 ^AD8–GFP^. GFP-expressing virions (yellow) were associated to migrated DCs.

To trace the virus in its path through the mucosa, human colonic explants were exposed for 30 min to R5 HIV-1^AD8^ or HIV-1^J6363^ or medium as control. We show that the mucus, which was still present in the explant culture substantiating the physiological relevance of the model, had trapped numerous virus particles (Fig 5B and C). Virus was also detected throughout the epithelial cell layer, and co-localized with DCs that migrated inside or beneath the epithelium (Fig 5B–D). Analogously, in the *in vitro* Caco-2/DCs culture model HIV-1^AD8^ was visualized on migrated DCs as soon as 30 min following virus incubation, and increased further after 4.5 h of incubation (Fig 5E–G). Viruses were detected associated to DCs' cell membrane as well as in the cytoplasm (Fig 5 and Fig S6 of Supporting Information). Some of the particles presented bulk and shapes, suggesting that they may be viral aggregates more than individual viral particles (Fig 5E and F). Only sporadically virions were found associated to non-migrated DCs, which were at the basal side (Fig 5F). GFP^+^ virions were also detected on DCs after detachment from the filter (Fig S6 of Supporting Information) by both CM and flow cytometry. Thus, we show that migrated DCs actively sample R5 HIV-1 both *ex vivo* and *in vitro*, providing proof of a new mechanism by which R5 viruses may get access to the intestinal mucosa.

### DC migration across the epithelial monolayer is mediated by viral envelope and CCR5

**Figure 6:**
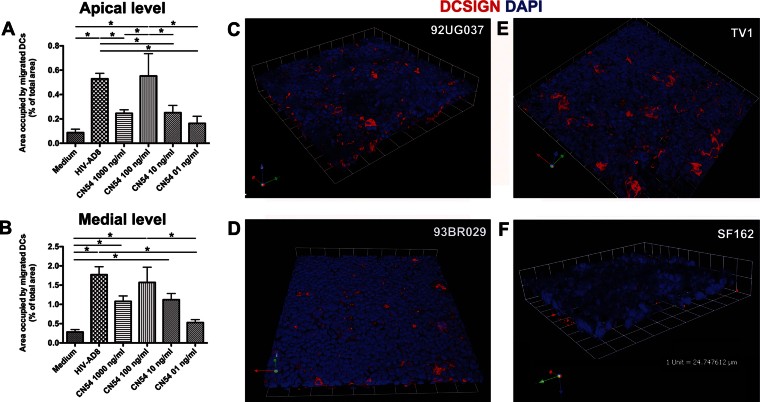
DC migration across the epithelial monolayer in response to gp140 protein of different HIV-1 subtypes. A,B. The monolayer of the Caco-2/DCs system was incubated for 1.5 h with decreasing dose of R5 CN54 gp140 trimeric protein. Medium and R5 HIV-1^AD8^ were used as negative and positive control, respectively. Bar charts represent quantitative analysis of DCs migration across the Caco-2 cell at the apical (A) and medial (B) level of the cell monolayer (as in Fig 1). Results are expressed as percentage of area occupied by DCs compared to that of the whole field. Bars represent mean ± SD of three different fields of three different experiments. *p < 0.05. C–F. DC migration was induced with gp140 protein (100 ng/ml) of 92UG037 (C), 93BR029 (D), and TV1 (E) but not with that of SF162 (F). Representative three-dimensional renderings from CM *z*-series stacks of the Caco-2 and DCs on filter stained for DAPI (epithelial cells and DCs; blue) and mouse anti-human DC-SIGN-PE (DCs; red) are shown. Results are those from one representative experiment out of three.

**Figure 7:**
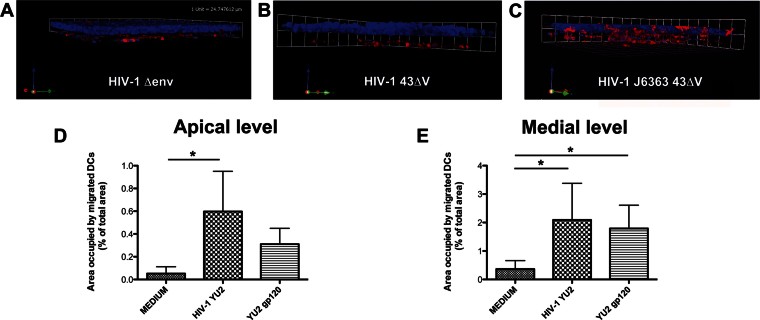
DCs migration is dependent from the viral envelope. In the absence of the HIV-1 env as well as of the V1V3 env region, DCs did not migrate across the Caco-2 monolayer (A and B). The V1V3 env region of an R5 virus completely restored the migratory ability of DCs (C). A–C. The Caco-2/DCs co-culture was incubated apically for 1.5 h with HIV-1 deleted of the env (HIV-1^Δenv^, 6 ng of p24 Ag) (A), the HIV-1^43ΔV^ deleted of the V1V3 region (40 ng of p24 Ag) as negative control (B) and a recombinant HIV-1 with the V1V3 env region of the R5 HIV-1^J6363^ primary virus recombined with the HIV-1^43ΔV^ backbone (HIV-1^J6363-43ΔV^, 20 ng of p24 Ag) (C). Three-dimensional reconstructions from CM *z*-series image stacks of the Caco-2/DCs culture stained for DAPI (epithelial cells and DCs; blue) and mouse anti-human DC-SIGN-PE (DCs; red) are shown. D,E. The monolayer of the Caco-2/DCs system was incubated for 1.5 h with medium (negative control), HIV-1^YU2^ (positive control) or YU2 gp120 protein (100 ng/ml). Bar charts represent quantitative analysis of DCs migration across the Caco-2 cell at the apical (D) and medial (E) level of the cell monolayer (as in Fig 1). Results are expressed as percentage of area occupied by DCs compared to that of the whole field. Bars represent mean ± SD of three different fields of three different experiments. Statistic analysis was performed as described in Materials and Methods Section. *p < 0.05.

Given the concordance between the results of the *in vitro* and *ex vivo* models, we adopted the *in vitro* Caco-2/DCs model to further investigate the mechanism involved in the migration event. The selective ability of R5 HIV-1 to trigger migration of DCs, prompted us to hypothesize the involvement of the viral envelope (env), which contains the major determinants for the specific binding to the chemokine receptors (Hwang et al, 1991). Indeed, the CN54 trimeric gp140 protein (subtype C and R5 phenotype) was able to induce in a dose-dependent manner the same migratory process of the DCs into the Caco-2 monolayer as the whole virus did. The cellular response to protein stimulation followed a Gaussian distribution with the highest effect observed when the protein was used at a concentration of 100 ng/ml (Fig 6A and B and Fig S7 of Supporting Information). Thus, we chose this concentration to test five additional R5 gp140 proteins of different genetic subtypes, *i.e.* TV1 (subtype C), 92UG037 (subtype A), 93BR029 (subtype F), YU2 and SF162 (both subtype B). All the trimeric gp140 proteins triggered DCs migration with the exception of SF162 gp140, even when added at doses as high as 5 µg/ml (Fig 6C–F and data not shown). Moreover, the YU2 trimeric gp120 protein alone as well as the X4 pseudovirus HIV-1^43ΔV^ when recombined with the V1V3 env region of the R5 HIV-1^J6363^ (HIV-1^J6363-43ΔV^) determined migration too (Fig 7), further indicating that the determinant(s) were within the gp120. More specifically, YU2 trimeric gp120 protein deleted of the third hypervariable (V3) loop had a substantially reduced ability to induce DC migration (mean 64 and 51% of reduction compared to wild-type gp120 at the apical and medial level, respectively). This data suggests that the V3 loop, which is considered to be the major viral determinant for coreceptor specificity, is fundamental to induce DCs translocation across the epithelium.

**Figure 8:**
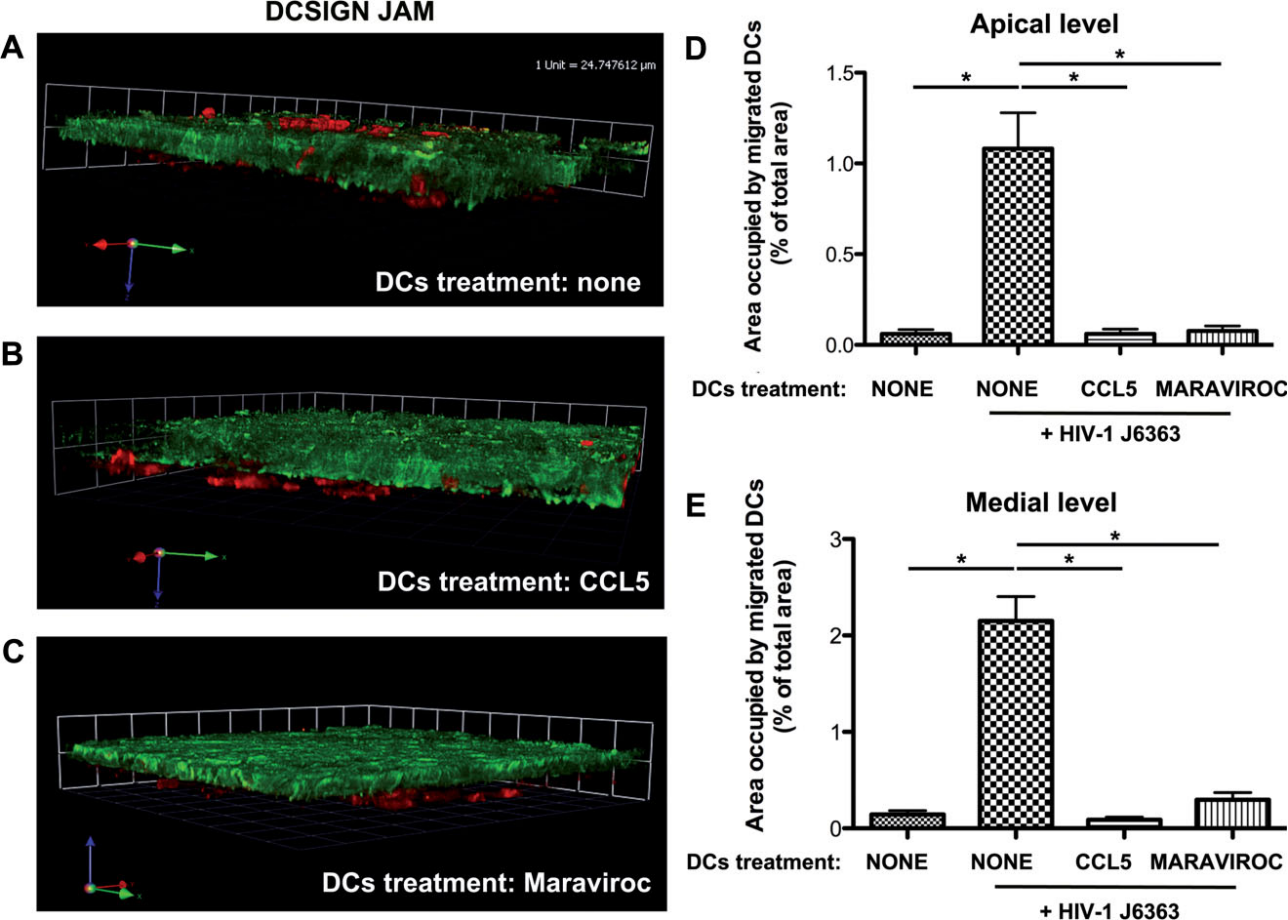
Engagement of CCR5 is necessary to trigger migration of DCs across the epithelium. A–C. DCs were left untreated (A) or were incubated for 4 h during attachment to the filter with CCL5 (200 ng/ml) (B) or Maraviroc (100 ng/ml) (C), and thereafter R5 HIV-1^J6363^ was incubated apically on the Caco-2 cells monolayer for 1.5 h. Representative three-dimensional rendering from CM *z*-series stacks of the Caco-2/DCs culture stained with mouse anti-human JAM + Alexafluor 488 goat anti-mouse IgG (epithelial cells; green) and mouse anti-human DC-SIGN-PE (DCs; red) is shown. Results are of one representative experiment out of three. D,E. Quantitative analysis of DCs migration across the Caco-2 cell monolayer at the apical (D) and medial (E) level of the cell layer revealed complete inhibition of DCs migration, induced by the virus, following CCL5 or Maraviroc treatment. Results are expressed as percentage of area occupied by DCs compared to that of the whole field. Bars represent mean ± SD of three different fields of three different experiments. *p < 0.05. In the negative control the Caco-2 cells monolayer was stimulated with medium only and DCs were left untreated (bar on the left in D and E).

The specificity of the R5 viruses prompted us to focus on CCR5 as a possible counterpart involved on the DCs. To down-modulate or otherwise merely occupy the CCR5 molecule, DCs were incubated for 4 h – during attachment to the filter – with the CCR5 ligand CCL5 (Fig S8 of Supporting Information) or the allosteric, non-competitive antagonist drug Maraviroc, respectively. HIV-1^J6363^ as well as HIV-1^AD8^-induced migration of DCs was clearly abrogated by both CCL5 and Maraviroc treatment (Fig 8), confirming the involvement of CCR5 in DCs recruitment. To exclude the possibility of an effect due to a reverse chemotactic gradient induced by the addition of CCL5 during attachment, DCs were treated with the chemokine for 2 h before attachment to the filter: again, virus-induced DCs migration was completely inhibited (data not shown). These findings indicate that HIV-1 induced migration of DCs is specifically mediated by the env glycoproteins' cognate coreceptor CCR5.

### HIV-1 enters inter-epithelial space without tight junctions disruption

The rapidity of the DCs migratory process hinted to the possibility, that chemokines secreted by Caco-2 cells upon viral stimulation, could drive the process. However, the concentration of the CCR5-binding chemokines CCL3, 4 and 5 in both apical and basal chamber supernatants of Caco-2/DCs culture, of the Caco-2 only culture, or in the cell lysates of Caco-2 cells after 1.5 and 24 h of incubation with R5 (HIV-1^AD8^ and HIV-1^J6363^) or X4 (HIV-1^pNL4.3^ and HIV-1^IIIB^) viruses, was below the detection limit of the technique. Instead CCL2 was absent when Caco-2 cells were cultured alone, but was detected in either chamber in the Caco-2/DCs cultures, however, at comparable levels in HIV-1- and medium-treated samples (65 ± 32.5 *vs.* 43 ± 21.5 pg/ml in the apical and 157 ± 149.2 *vs.* 115 ± 69.3 in the basal supernatant, respectively). CCL20, a chemokine known to be produced by both epithelial cells and DCs, was detected exclusively in the apical chamber of both Caco-2 cells and Caco-2/DCs cultures after 24h of incubation, but with no significant difference between HIV-1 and medium-treated samples (Caco-2 cells alone: 77 ± 42 and 86 ± 15 pg/ml; Caco-2/DCs: 65 ± 6.5 and 65 ± 12.6 pg/ml in the medium and HIV-treated samples, respectively). These data clearly show that under these specific culture conditions Caco-2 cells and/or DCs produce CCL2 and CCL20 but that the event is independent from the virus.

We also excluded that chemokines present in the viral inoculum may determine DCs migration. Supernatants of the PBMC-based cultures of either viral phenotype, *i.e.* HIV-1^J6363^ and HIV-1^IIIB^, contained CCL3, 4 and 5 at comparable concentrations (Table 1), whereas those of R5 and X4 viruses produced in 293T cells, had levels below the detection limit of the technique. CCL2 and CCL20 were not detected in viral culture supernatants. Although we did not detect the chemokines we cannot exclude that a localized production and accumulation at the Caco-2 cell membranes occurred, which could nevertheless play a role.

**Table 1:** Chemokine concentrations of viral culture supernatant

HIV-1 supernatant	Viral culture	CCL3 (pg/ml)	CCL4 (pg/ml)	CCL5 (pg/ml)
HIV-1^AD8^	293T cells	<16^a^	<6^a^	<3^a^
HIV-1^J6363^	PBMC	1590	1099	315
HIV-1^pNL4.3^	293T cells	<16^a^	<6^a^	<3^a^
HIV-1^IIIB^	PBMC	653	271	526

CCL3, 4 and 5 levels in HIV-1 culture supernatants were measured with Luminex (R&D system).

a Value below the lower detection limit of the assay.

**Figure 9:**
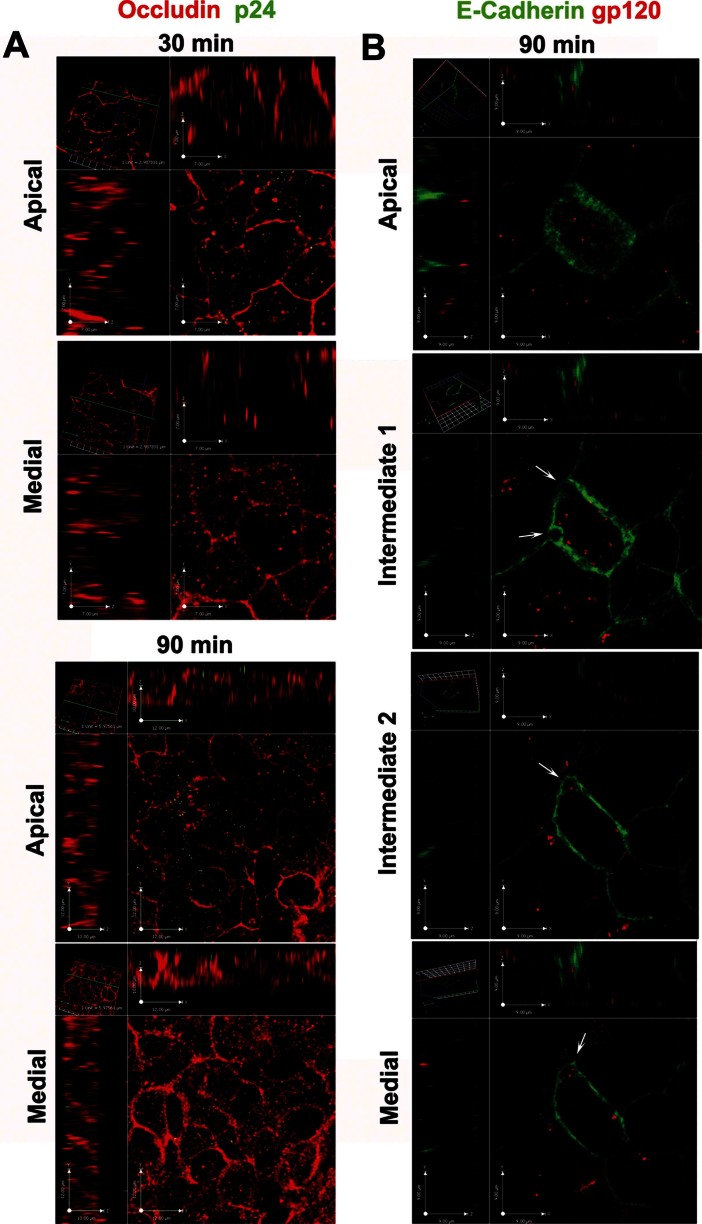
HIV-1 penetrates within and in between epithelial cells. A. Virions (stained with mouse anti-p24 + Alexafluor488 goat anti-mouse IgG, green) were mainly localized at the apical surface of Caco-2 cells at 30 min but also inside the cytoplasm at 90 min of incubation with R5 HIV-1^AD8^ (20 ng of p24 Ag). Shown are CM single plane cross sectional images of a Caco-2 monolayer (rabbit anti-human Occludin + Alexafluor594 goat anti-rabbit IgG; red) taken at apical and medial level of the cell layer. B. R5 HIV-1^AD8^ (red; visualized with human anti-gp120 monoclonal antibody 2G12 + Alexafluor594 goat anti-human IgG) localized inside the cytoplasm of epithelial cells and in intrajunctional spaces (indicated by arrows). Shown are four single plane cross sectional images, taken from the apical to the medial plane along the *z*-axis of the Caco-2 monolayer (mouse anti-human E-Cadherin + Alexafluor488 rabbit anti-mouse IgG2a; green) incubated with R5 HIV-1^J6363^ (at 5 ng of p24 Ag) for 90 min. Results are of one representative experiment out of three.

To further investigate if the virus itself could be an active player, we traced its passage throughout the Caco-2 monolayer by different means. Viruses (HIV-1^AD8^ and HIV-1^J6363^) were increasingly bound to and internalized in Caco-2 cells in a time-dependent manner without altering the integrity of the monolayer (Fig 9A, and Fig S9 of Supporting Information). In addition, we detected viruses also in between adjacent epithelial cells in intrajunctional spaces localized closely to the adherent junctions (Fig 9B), despite the apical TJs were still present and tightly adherent. This suggests that HIV-1 may access the intercellular space either inducing a transient opening of TJs to enter between adjacent cells or entering the epithelial cells and then being released from the cytoplasm laterally into the basal intercellular space. In either case, virions may accumulate between cells and conceivably create an intercellular gradient, which in turn may act as a chemoattractant and promote DCs migration.

### Trancytosis *per se* is not sufficient to engage DCs

**Figure 10:**
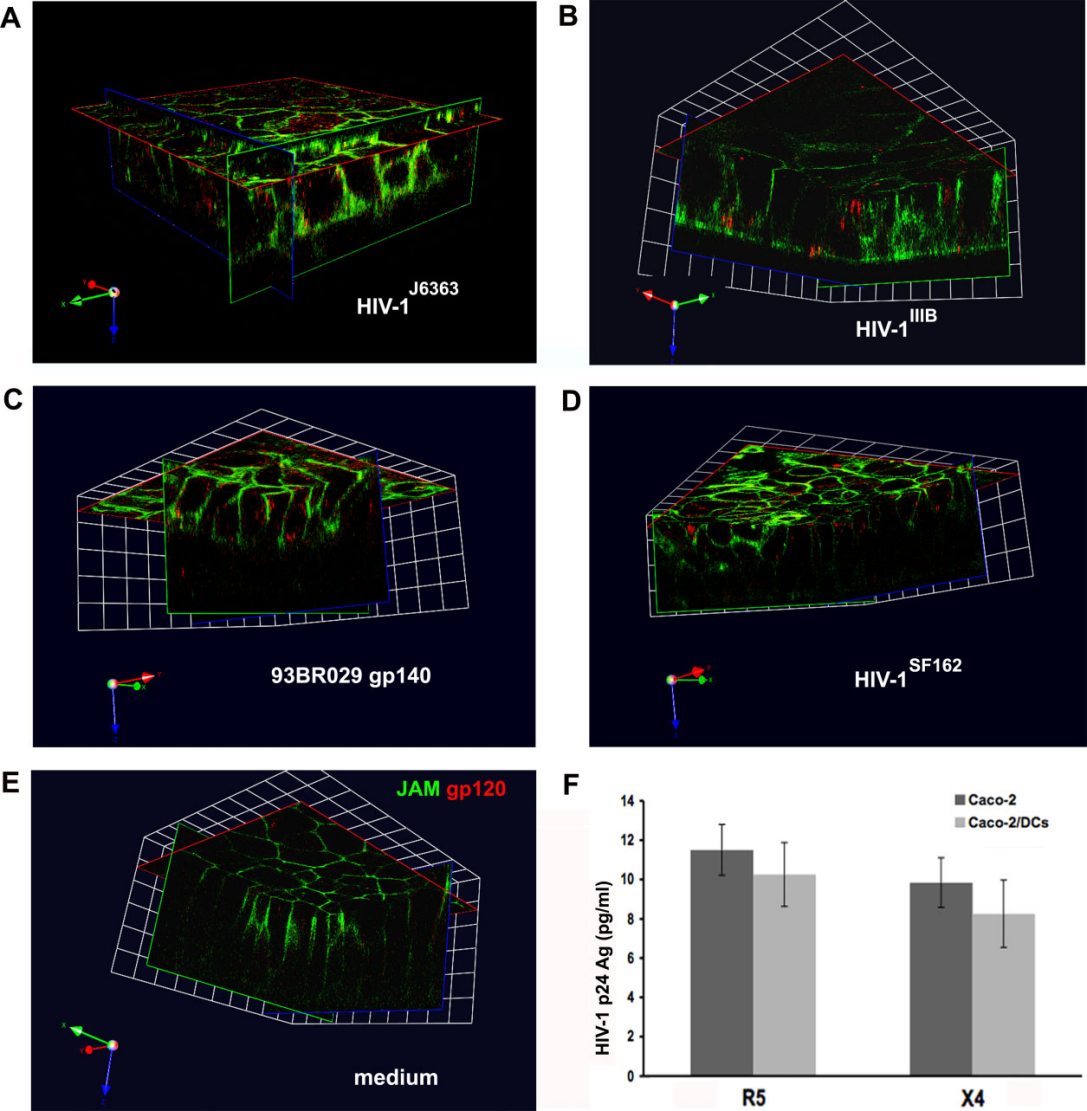
Transcytosis of cell-free HIV-1 through a tight monolayer of Caco-2 cells. A–E. R5 HIV-1^J6363^ (A, at 1 ng of p24 Ag), X4 HIV-1^IIIB^ (B, at 10 ng of p24 Ag), R5 93BR029 gp140 protein (C, at 100 ng/ml), R5 HIV-1^SF162^ (D, at 2 ng of p24 Ag), or medium (E), were added to the Caco-2/DCs cultures for 90 min and immunostained for CM analysis. HIV-1 (red; visualized with human anti-gp120 monoclonal antibody 2G12 + Alexafluor594 goat-anti-human IgG) was dispersed throughout the cytoplasm but mainly concentrated in the upper part of the Caco-2 cells (visualized with mouse anti-human JAM + Alexafluor 488 goat-anti-mouse IgG; green). DCs are not shown in the Figure. Transversal *xy*- and *xz*-plane visualization from representative fields of the monolayer were obtained with Volocity 5.0 software. 1 unit = 10.3 µm. Results show one representative experiment out of three. F. Transcytosis of HIV-1 R5 and X4 is comparable. The amount of transcytosed virus was evaluated measuring with ELISA the p24 Ag released in the basal chamber after 2.5 h of incubation with cell-free HIV-1 (at 20 ng of p24 Ag), either R5 HIV-1^AD8^ or X4 HIV-1^pNL4.3^, on the apical side of the Caco-2 monolayer cultured with or without DCs adherent to the filter. Results are expressed as mean ± SD of triplicates of three different experiments.

As transcytosis is a recognized pathway used by HIV-1 to get access to the intestinal lamina propria (Bomsel, 1997), we asked the question whether the transcytosed virus was sufficient to trigger DCs migration across the epithelium. The immunofluorescence staining of the viral *env* gp120 with the 2G12 monoclonal antibody inside the Caco-2 cells exposed to viruses of either phenotype, confirmed that both R5 and X4 viruses enter and pass through the cytoplasm of epithelial cells (Fig 10A–E). Surprisingly, also the env of HIV-1^SF162^, which does not induce migration, was detected within the cytoplasm of Caco-2 cells (Fig 10D), proving that transcytosis *per se* is not sufficient to trigger DC migration.

The amount of transcytosed virus was comparable for R5 and X4 HIV-1, *i.e.* the p24 Ag levels detected in the basal medium were an average of 11.5 and 9.8 pg/ml after 2.5 h of incubation with the pseudoviruses HIV-1^AD8^ and HIV-1^pNL4.3^, respectively (Fig 10F). In addition, the presence of DCs in the culture system did not alter the transcytosis efficiency, as similar levels of transcytosed virus were measured. PHA-activated PBMCs placed in the basolateral chamber of the *in vitro* model in the presence or absence of DCs, actively replicated the transcytosed virus, thus supporting that the transcytosed virus is infectious (Table S1 of Supporting Information).

The kinetic experiments performed incubating R5 HIV-1^AD8^ and X4 HIV-1^pNL4.3^ on the apical side of Caco-2 cells at regular time intervals between 30 min and 4.5 h confirmed previous published data (Bomsel, 1997), that transcytosis is time-dependent and reaches a peak after approximately 3 h independently of the viral phenotype (Table S1 of Supporting Information), being, thus, a delayed process and not selective for R5 viruses compared to DCs migration.

### DCs efficiently transmit HIV-1 to target cells

**Figure 11:**
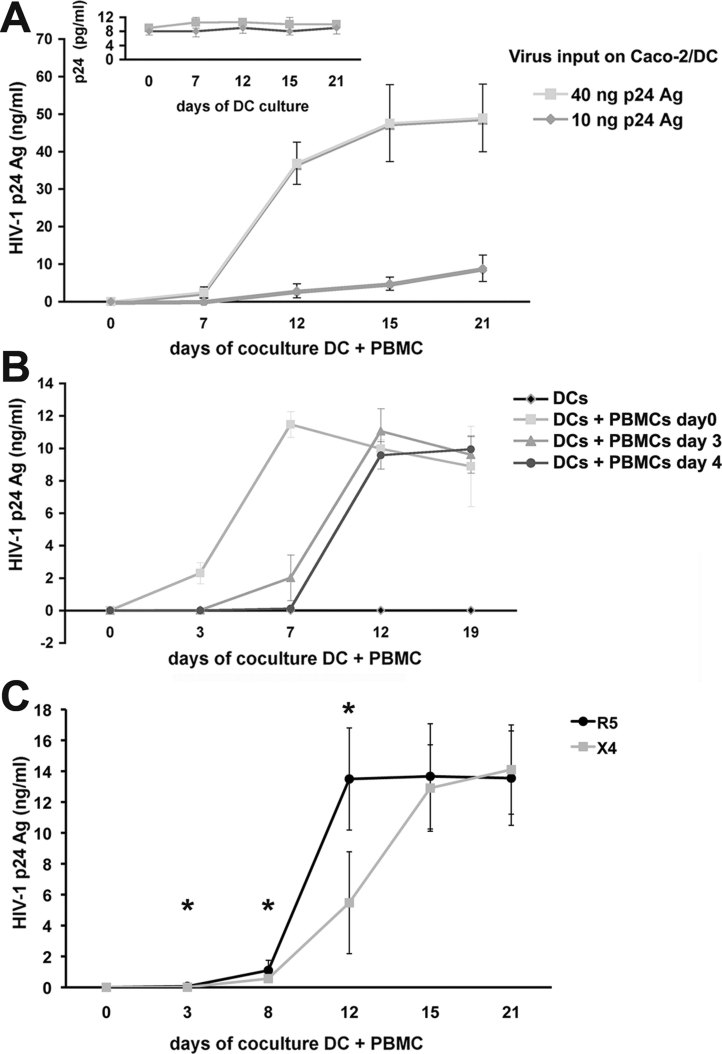
DCs capture virus and transfer infection to target cells. A,B. Caco-2/DCs system was apically incubated for 1.5 h with R5 HIV-1^AD8^ at 10 or 40 ng (A) or 20 ng of p24 Ag (B). PBMCs (5 × 10^5^ cells) directly incubated with the same amount of input virus served as positive control (data not shown). (A) R5 virus does efficiently replicated when DCs were cultured with PBMCs (1 × 10^6^). Low levels of p24 Ag were observed when DCs were cultured alone (inset). (B) DCs transmit infectious virus in the long-term. PBMCs were added to detached DCs immediately or after 3 or 4 days. Results from a representative experiment out of three are expressed as mean of p24 Ag values of triplicate cultures ± SD. C. DCs transferred both R5 and X4 viruses to PBMCs. Caco-2 cells were treated with R5 HIV-1^AD8^ (24 ng/ml) and X4 HIV-1^pNL4.3^ (40 ng/ml) as described above. DCs collected from transwell were co-cultured with PHA-activated PBMCs. Mean ± SD of three different experiments performed in triplicate is shown. *p < 0.05.

To further define the functionality of DCs, we investigated whether DCs detached from the filter after incubation with HIV-1^AD8^ for 1.5 h on the apical side of Caco-2 cells were able to replicate the virus and to transfer infection to HIV-1 target cells. After detachment, DCs when cultured alone showed constant and persistent low levels of virus release (6–12 pg/ml of p24 Ag) for up to 3 weeks (inset in Fig 11A), which may not necessarily represent active viral replication. However, virus production became considerable when DCs were co-cultured with PBMCs (Fig 11A), and was dependent from the concentration of virus inoculum used (Fig 11A). Furthermore, virus could also be recovered when PBMCs were added to the DC culture after 3 or 4 days (Fig 11B), thus demonstrating that DCs remained infectious in the long-term.

We further traced the fate of the virus in the DCs by concomitant incubation with fluorescent ovalbumin and labelling with Lyso Tracker to discern the localization in acidic lysosomes. After 1.5 h of incubation with HIV-1^AD8GFP^ about 60% of DCs harboured the virus, which was evenly distributed at the level of the cell membrane (Fig S10 of Supporting Information). Thereafter, at 30 min, most of the virus was within the cells but did not co-localize with neither ovalbumin-positive vesicles nor lysosomes. At 6 h viruses detected in less than 10% of cells, had accumulated in a perinuclear area. After 24 h, no virus was detected, which was consistent with a previous report (Yu et al, 2008). These results taken together with the recovery of virus by addition of PBMCs to DCs after 4 days in culture, suggest that the virus was also integrated in DCs.

As we showed that both virus phenotypes passed through the epithelium the infection of DCs by X4 viruses was studied. The X4 virus HIV-1^pNL4.3^ replicated in the coculture of detached DCs with PBMCs (Fig 11C) with a slower kinetics than the R5 virus to reach similar levels of p24 Ag production in the long-term. Thus, we showed that viruses with either phenotype can enter the mucosal epithelial surface to reach DCs, which remain infectious for prolonged time and may act as reservoir.

## DISCUSSION

HIV-1 can be detected in body fluids such as breast milk or seminal plasma despite control of the viral load in the blood with effective ART (Neveu et al, 2011; Zhang et al, 1998) and provides a continuous source of virus particles to easily spread the infection. DCs or Langerhans cells of the intestinal and vaginal mucosa were repeatedly described to be the first target for HIV-1 and the vehicle for the virus to reach replication competent cells (Hladik et al, 2007; Hu et al, 2000; Shen et al, 2010; Spira et al, 1996). Whether DCs/Langerhans cells have an active role in capturing the virus through the epithelial cell layer or are infected following viral release into the lamina propria, can have important implications in prevention strategies. Here, we finally demonstrate that DCs rapidly migrate through a monostratified intestinal epithelium only in response to CCR5-using HIV-1, independently of genetic subtype, in an *ex vivo* organ culture and an *in vitro* Caco-2/DCs polarized culture. Whether such mechanism of DCs migration occurs also in pluristratified epithelia will require further analysis. We provide evidence that DCs migration across a tight intestinal epithelium is triggered by the R5 viral env itself engaging DCs via the CCR5 molecule. Accordingly, DCs migration is abrogated by treatment of the cells with CCR5-binding ligands. These results further support the use of CCR5 inhibitors for prevention, as already suggested by the Rhesus Macaques vaginal infection model, where vaginal application of CCR5 inhibitors prevented transmission of chimeric Simian-HIV (Lederman et al, 2004; Veazey et al, 2005). It is conceivable, that analogous interventions can be implemented also for prevention of infection of rectal tissue, where drugs were shown to be more efficient than in vaginal tissues (Herrera et al, 2011).

The migration process of DCs is R5 viral env mediated and is further restricted to the V3 loop, suggesting that an env-CCR5 interaction is necessary to initiate DCs migration across the epithelium. Interestingly only one (SF162) out of 6 R5 viruses was not able to trigger migration but was transcytosed through epithelial cells. If this is an exception to the rule or if other viruses may have its same restrictions will need further testing and possibly help to identify specific determinants. In addition, viral envelopes with the intrinsic characteristic to induce migration of and be captured by DCs may be selected and exploited in mucosal vaccination strategies to favour antigen presentation.

The thorough tracing of the virus travel through the epithelial monolayer revealed that the release of virus through a transcellular pathway was not sufficient to engage DCs and, that the virus can penetrate in between adjacent epithelial cells and localize in intrajunctional spaces close to the adherent junctions. We propose that HIV-1, similarly to other viruses (Nava et al, 2004; Wang et al, 2011), induces a transient opening of the TJs to gain access through the epithelium. It may be plausible that intraepithelial virions can be also released laterally into the interepithelial spaces and thus, create a paracellular viral gradient attracting DCs. The role of the virus itself is also supported by the absence of detectable levels of CCR5-binding chemokines in the culture systems together with the previously published observation that HIV-1 R5 env is chemotactic for DCs (Lin et al, 2000). Analogously it was described that HIV-1 gp120 binding to CCR5 activates a signaling cascade leading to phosphorylation of the nonreceptor tyrosine kinase Pyk2, and downstream activation of p38MAPK, which ultimately leads to DCs migration (Anand et al, 2009; Harman et al, 2006; Wilflingseder et al, 2004). Thus, it is conceivable that also in our model signaling through CCR5 may be required to induce DCs migration across the epithelium.

We observed the formation of junction like structures between DCs and epithelial cells, which may initiate the haptotaxis of DCs, namely their directional motility towards a gradient of cellular adhesion sites. We showed previously that blocking the TJs proteins on DCs inhibits the opening of adjacent epithelial cell junctions (Rescigno et al, 2001; Rimoldi et al, 2004). We may envisage that the close contact between DCs and epithelial cells may also favour cell-to-cell viral spreading of intraepithelial virions. It is tempting to speculate that during transmission of mixed R5 and X4 viruses these latter ones, internalized in the epithelial cells and unable to attract DCs themselves, may hijack migrated DCs for their own purpose.

Some studies showed a decreased barrier function and enhanced permeability following HIV exposure using *in vitro* cultures of intestinal epithelial cells (Nazli et al, 2010; Schmitz et al, 2002). We provide several evidences that in our experimental setting the epithelial barrier is instead completely preserved. This discrepancy could be ascribed to different factors: we used lower virus input and shorter time of virus exposure compared to the other studies, which makes our experimental setting closer to physiological conditions. However, also in our hands the morphology of the epithelial monolayer is altered with long-term exposure, which underlines the necessity of severely controlled settings in the mucosal culture systems. In our *ex vivo* organ culture, we confirmed that the virus is heavily trapped by the mucus present on the epithelial cells, as previously shown (Maher et al, 2005). It would be relevant to understand how infected body fluids, such as milk and semen, may impact on epithelial integrity or cellular cross-talk. Indeed, some studies indicated that seminal plasma can lead to inflammatory response and facilitate HIV-1 transmission (Planchon et al, 1999), other studies showed instead an enhanced epithelial barrier function mediated by seminal plasma components (Gorodeski & Goldfarb, 1998). Unfortunately, the frailty of these organ culture models does restrict very much its use.

DCs fail to migrate in response to X4 viruses both in the *in vitro* and *ex vivo* culture models used in our study. In the *in vitro* system, it may be ascribed to low levels of CXCR4-expression on DCs used, however, we show that DCs expressing a comparable level of the two co-receptors remain unresponsive to X4 viruses. In human colon, DCs express constitutively CXCR4 and CCR5 (68.6 ± 16.7 and 36.7 ± 19.35%, respectively). It can be envisaged, that the stromal derived factor-1, which is expressed by intestinal epithelial cells (Agace et al, 2000), may interphere with the binding of the virus to CXCR4, thereby preventing the migration of DCs in tissues.

We show with both models that DCs migrate and capture R5 virus as fast as within 30 min, which indicates that they are the first cells that capture HIV-1 in the intestine. However, the extent to which DCs in the intestine are infected is still unknown and would need a thorough analysis. Interestingly, the migration process is specific for lamina propria resident DCs and not Mϕ, which instead remained confined within the subepithelial layer. An involvement of Mϕ in the subsequent passage of HIV-1 within the intestinal mucosa may not be excluded, however, human intestinal Mϕ were shown to be poorly infected (Smith et al, 1994), and did not support efficient viral replication *in vitro* (Li et al, 1999; Meng et al, 2000; Shen et al, 2011). Furthermore, in contrast to Mϕ, DCs can migrate into draining lymph nodes and thus, may play an active role in viral spreading from the very first moments after infection.

Interestingly, we showed that DCs migration is reversible, thus DCs sensing a chemokine gradient may migrate back into the lamina propria to further spread infection. This observation is in line with a recent study showing that in a penile foreskin culture LCs move from the epidermis to the dermis after 4 h from exposure to HIV-1 infected PBMC and form conjugates with T cells (Zhou et al, 2011). The relevance of DCs in disseminating further the virus, as early as 24 h after infection, to genital lymph nodes was demonstrated in intra-vaginally inoculated animals (Hu et al, 2000; Masurier et al, 1998; Miller et al, 1994). The question if virus loaded DCs of the intestine will also migrate to the lymphatic system or just to the lamina propria cannot be concluded *in vitro* but will need *in vivo* studies. In the mouse intestine, it was described that CD103^+^ DCs migrate to lymph nodes whereas the CX3CR1^+^ ones are deputed to extend dendrites to sample bacterial antigens in response to fractalkine released by the epithelium (Niess et al, 2005; Schulz et al, 2009). The DCs used in our *in vitro* model express CX3CR1, but their migration was not abrogated by pre-treatment with its ligand fractalkine (data not shown). Thus, it will be interesting to understand whether these two functionally distinct sub-sets of antigen presenting cells have also in human similar functions.

There is plenty of evidences that to spread the infection the DCs transfer virus to CD4^+^ T cells, which sustain actively viral replication, via both vesicular uptake (called *trans* infection) and *de novo* production (called *cis* infection) (Garcia et al, 2008; Geijtenbeek et al, 2000; Hladik et al, 2007; Hu et al, 2004; Pope et al, 1994; Turville et al, 2004). Here, we show that also those DCs, which sampled virions through an epithelial barrier, maintain the ability to spread the infection to CD4^+^ T cells for at least 4 days from infection. These same DCs, when cultured alone, were showing low levels of virus release, which may either represent active viral replication or release of membrane-attached virions. R5 HIV-1 was rapidly internalized by DCs and redirected into an intracellular compartment, which neither co-localized with ovalbumin-positive retention vesicles nor with acidic lysosomes. However, the GFP^+^ virus was lost within 24 h suggesting that it did not remain intact inside the cells. Our observation that transfer to CD4^+^ T cells could occur after several days of culture, indicates that the pathway we observed lead, at least in part to virus integration. The low expression of CXCR4 on DC would instead hamper their direct infection and favour the *trans* pathway. These results are in line with previous reports suggesting a bi-model transmission of virus (Cavrois et al, 2007; Turville et al, 2004), *i.e.* in the short period virus was transferred in *trans* whereas in the long period through *de novo* production. In conclusion, our data indicate that DCs can act as reservoir and become a source of virus detrimental for the surrounding cells and affecting innate and adaptive immune responses.

**Figure 12:**
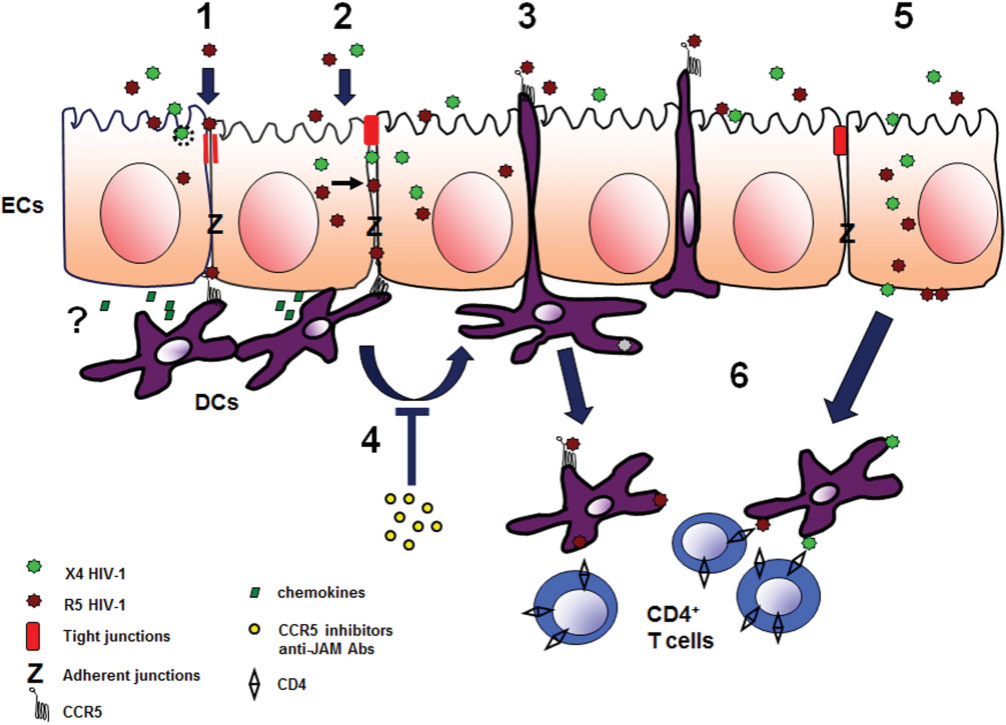
Schematized proposed mechanism of HIV-1 penetration across the intestinal epithelium and DCs recruitment. HIV virions/gp120 env protein that interact with the apical surface of intestinal epithelial cells (ECs) can transiently open the TJs of EC (1) or enter into the EC and be released from the cytoplasm to the intercellular space (2) to create a gradient between adjacent EC and reach subepithelial DCs. Chemokines secreted by EC might possibly accumulate locally. The interaction between the env and the CCR5 receptor expressed on DCs induces within 30 min the elongation of DC's dendrites and migration of DCs across the epithelium to further capture luminal R5 virions (3). The process is inhibited by CCR5 inhibitors and anti-JAM antibodies (Abs) (4). Alternatively, virions can be transcytosed through ECs (5) and be captured by lamina propria cells. Migrated DCs can shuttle across the barrier and return to the lamina propria when appropriately stimulated. The captured virus is transmitted from DC to CD4^+^ T cells (6) or directly transported to lymph nodes to further spread the infection.

In Fig 12, we present a schematic model of the different pathways used by R5 and X4 viruses to access the intestinal mucosa and disseminate within the host. Despite most of the mechanisms used are similar for the two viral phenotypes, R5 viruses are the only capable of selectively recruit DCs to migrate through the epithelium to capture luminal virions. This pathway, which may possibly give to R5 viruses an advantage in migrating to lymph nodes to further spread the infection, could be targeted to design new prevention strategies.

## MATERIALS AND METHODS

### Ethics statement

The use of colonic tissues and HIV-1^J6363^ primary isolate was approved by the Ethical Board of the San Raffaele Scientific Institute. A written consent was obtained from patients.

### Cells, viruses and reagents

Immature DCs were obtained from CD14^+^ cells isolated from PBMC and cultured for 5–6 days in RPMI 1640 with 1% FCS, 50 ng/ml granulocyte–monocyte colony-stimulating factor and 20 ng/ml Interleukin-4 (Peprotech Inc., NJ). DCs showed a high expression of DC-SIGN, CD1a and CD11c, low expression of HLA-DR, CD80 and CD83 and a minimal expression of CD14 at flow cytometric analysis. DCs had significant expression of CD4, CCR5 and CX3CR1, but minimal (<2%) expression of CXCR4. For specific experiments, which needed DCs expressing similar level of CCR5 and CXCR4, cells were cultured for additional 16 h with “colon-conditioned media”, produced as described in the paragraph “Human colonic tissue *ex vivo* culture”.

Caco-2 cells (clone E) were grown in Dulbecco's modified Eagle's medium (DMEM) with 10% FCS, 1% non-essential amino acids and penicillin (100 U/ml)/streptomycin (100 µg/ml). All reagents were purchased from Lonza Group (Switzerland) unless otherwise indicated.

HIV-1^J6363^ primary isolate (R5 phenotype and subtype B) was obtained from an HIV-1 infected child. HIV-1^J6363^, HIV-1^SF162^ and HIV-1^IIIB^ virus stocks were produced in PHA-stimulated PBMCs, as previously described (Scarlatti et al, 1991). Pseudoviruses were produced transfecting 293T cells with pAD8, pNL4.3 or pNL4.3Δenv plasmids (provided by Dr. Bovolenta) to generate HIV-1^AD8^, HIV-1^pNL4.3^ and HIV-1^Δenv^ or with E7-HIV-1-YU2gp160 expressing plasmid (provided by Dr. Sodroski) together with pSG3Δenv to produce HIV-1^YU2^ using Fugene 6 (Roche, Indianapolis, IN, USA). GFP-Vpr-labelled HIV-1^AD8^, designated as HIV-1^AD8GFP^ throughout the manuscript, was produced by transfection of 293T cells with the proviral construct pAD8 and the plasmid pGFPC3 (kind donation of Dr. Paxton) containing the entire Vpr coding region fused to the carboxy-terminus of eGFP (GFP-Vpr) as previously described (McDonald et al, 2002). Recombinant viruses HIV-1^43ΔV^ and HIV-1^J6363-43ΔV^ were produced as previously described (Trouplin et al, 2001). Briefly, 293T cells were transfected with 43ΔV vector (a pNL4.3 variant deleted of V1V3 region) either alone to generate HIV-1^43ΔV^ or with PCR amplified env region from J6363-infected PBMCs to generate HIV-1^J6363-43ΔV^. National Institute for Biological Standards and Control (Hertfordshire, UK) provided TriMab and gp140 proteins CN54, 93BR029 and 92UG037. Novartis Vaccine and Diagnostics (Cambridge, MA) provided gp140 proteins SF162 and TV1. Wild type and deltaV3 YU2 gp120 proteins were a gift of Dr. Wyatt. Lipopolisaccharide (LPS) was from Sigma–Aldrich (St. Louis, MO). CCL5 was a gift of Dr. Vangelista, and Pfizer (Pfizer Italia Srl) kindly provided Maraviroc. Viral proteins and culture supernatants were all tested with the Limulus amoebocyte lysate (LAL) assay (detection limit, 0.03 endotoxin unit/ml; Pyrogent Plus, U.S.) to exclude the presence of bacterial endotoxin.

### Caco-2/DCs *in vitro* co-culture system, HIV-1 capture by DCs and transcytosis assay

The Caco-2/DCs co-culture system was developed modifying a published protocol (Rescigno et al, 2001). Briefly, Caco-2 cells (1 × 10^5^) were seeded on the upper face of a 6.5-mm filter (3-µm pore Transwell filter, Costar, Cambridge, MA) in a 24-well plate for 7–8 days until a transepithelial resistance (TER) ≥330 Ω/cm^2^ was achieved. DCs (4 × 10^5^) were seeded on the opposite side of the filter for 4 h. When indicated, DCs were treated with the CCR5 ligand/inhibitor CCL5 (200 ng/ml) or Maraviroc (100 ng/ml) for 2 or 4 h. The different treatments did not impact on the attachment of DCs to the filter as verified at CM. Cell-free HIV-1 supernatants, LPS (from *Salmonella enterica* serotype Minnesota, 1 µg/ml, positive control), or soluble gp140 proteins were added on the apical side of the Caco-2 cells for 1.5 h unless otherwise indicated. Negative controls were medium (DMEM 10% FCS), supernatant from mock-transfected 293T cells or from a mock PBMCs culture. For evaluation at CM or TEM Caco-2/DCs filters were either fixed in 2% paraformaldehyde in PBS (PFA) or in 2.5% gluterahldehyde in cacodylate buffer respectively.

To evaluate reverse migration HIV-1 or medium were incubated for 2 h on the apical side of Caco-2/DCs system. After extensive washing, the transwell was incubated for 3 h in a new plate containing either medium or fractalkine (100 ng/ml) in the basal chamber. Sample were fixed with 2% PFA and analysed at CM.

To determine the number of DCs released in the apical culture chamber, DC were labelled with CSFE (Invitrogen) 5 µM 10 min at 37°C, washed and then let to adhere to the basal side of Caco-2 cells as in standard conditions. Following 2 h incubation with HIV-1 or medium on the apical side of the Caco-2 cells the apical media was collected, centrifuged and the number of CSFE^+^DCs determined by flow cytometry.

To determine the capture of virus DCs were detached from the filter and cultured either alone or with PHA-activated PBMCs from 2 donors (5 × 10^5^). Culture supernatants were collected at consecutive time points up to 21 days and viral growth determined by an HIV-1 p24 Ag ELISA (lower detection limit 4 pg/ml, Aalto Bioreagents, http://www.aaltobioreagents.ie).

In the transcytosis assay, cell-free HIV-1 or medium (negative control) was incubated on the apical side of the Caco-2 monolayer and the amount of transcytosed virus evaluated in the basolateral medium with p24 Ag ELISA. To determine whether transcytosed HIV-1 was infectious, transcytosis assay was performed in presence of 1 × 10^6^ PHA-activated PBMCs in the basal chamber. After transcytosis the apical chamber was removed and PBMCs were cultured for additional 7 days to evaluate replication of transcytosed virus using a p24 Ag ELISA.

### Quality control of the Caco-2 monolayer

The development of a tight monolayer was confirmed by confocal laser microscopy of TJ molecules and by the absence of the diffusion into the basal chamber of a solution of dextran-FITC (FD4, 250 µg/ml, 4 kDa; Sigma–Aldrich, St. Louis, MO) in the presence of medium, virus culture supernatant (HIV-1^AD8^, 120 ng/ml of p24 Ag) or CN54 gp140 protein (100 ng/ml) added in the apical chamber for 1.5 h of incubation at 37°C.

### Chemokine/cytokine quantification

The supernatants of the pseudovirus-produced or PBMC-cultured viruses as well as the Caco-2 lysates and supernatants of the transwell upper and lower chambers from cells incubated either with HIV-1 or medium were collected at 1.5 and 24 h, and were analysed for the presence of CCL2 and CCL20 by ELISA and CCL3, CCL4 and CCL5 by Luminex (from R&D system, Minneapolis, MN, detection limit 16, 16, 16, 6 and 3 pg/ml, respectively) according to manufacturers' instructions. Specimens were in triplicate and the experiments were repeated three times.

### Human colonic tissue *ex vivo* culture

The Department of Surgery provided intestinal colonic fragments from patients (*n* = 5) undergoing surgery for non-invasive colon cancer. Specimens were surgical leftover processed for *ex vivo* culture within 30–45 min from the excision. Their morphological preservation was verified by histology (standard Hematoxylin & Eosin) on randomly chosen fragments.

The colon explants were harvested in 0.9% saline solution containing antibiotics (100 U penicillin/ml and 100 µg streptomycin/ml, 50 mg/ml gentamicin). After abundant washes in the same solution the specimens were transferred into complete medium (RPMI supplemented with 10% FCS, 100 U/ml penicillin/streptomycin, 1% glutamine, 1% NEAA, 1% Na-pyruvate, 1% HEPES buffer 1 M) and the epithelial surface exposed. Small tissue cylinders (diameter 8.0 mm) including epithelium and submucosa were cut with a biopsy punch (Stiefel, Laboratories, Inc. North Carolina), and the tissue fragments placed on a sponge support (sponge area = 2.25 cm^2^, BioOptica, Milan, Italy) with the submucosa facing the sponge. A polystyrene cylinder (I.D. × H 4.7 mm × 8 mm, Sigma–Aldrich, St. Louis, MO) was sealed with veterinary glue (3M Vetbond, St. Paul, MN) onto the apical surface of the mucosa to avoid leakage. Specimens were placed in a 60 mm center-well organ culture dish (BD Falcon, San Diego, CA) containing 1 ml of complete medium.

Mucosal explant cultures were treated apically with viral culture supernatant (HIV-1^J6363^, HIV-1^AD8^, HIV-1^pNL4.3^ or HIV-1^IIIB^) or medium (negative control). After 30 min the apical medium was discarded, whereas the basal medium was collected and analysed for p24 Ag concentration. Absence of p24 Ag in the basal medium, determined with ELISA, confirmed the seal integrity of the tissue culture system without any viral leakage. After abundant rinse tissues were fixed in 4% PFA over night, cryoprotected in 10% sucrose, embedded in killik (BioOptica, Milano, Italy) and snap-frozen. At least 5–6 successive 10 µm-thick sections at a distance of 100 µm from each other obtained with Leica CM1850 cryostat (Leica Microsystems GmbH, Wetzlar Germany) were submitted to immunofluorescence.

To obtain “colon-conditioned media” colon tissue explants were cultured 16 h at 37°C at high oxygen (Tsilingiri et al, 2012) and thereafter the basal supernatant was collected, filtered and stored at −80°C.

**The paper explained**

**PROBLEM**

The gastrointestinal tract is the portal through which HIV-1 enters the host during mother-to-child and sexual-anal transmission. The virus, which use the CCR5 receptor (called R5) besides the CD4 molecule to enter the cells are more frequently transmitted than those using the CXCR4 receptor (called X4). Among the several cell types located in the mucosa, like CD4^+^ T lymphocytes, macrophages and DCs, the latter ones were shown to be one of the first targets for the virus that enters the intestinal lamina propria through an intracellular pathway called transcytosis. In this paper, we address the question if DCs have also a direct role in the translocation of the virus across the intestinal epithelium as demonstrated for other pathogens.

**RESULTS**

Here, we show, using *in vitro* model of intestinal mucosa and *ex vivo* human colon explant, that HIV-1 of R5 but not X4 phenotype selectively induces the migration of DCs across the intestinal epithelium. DCs sample luminal virions, retain the virus in the long-term and thereafter transmit infection to receptive target cells. Conversely, lamina propria macrophages were not able to migrate across the intestinal epithelium in response to HIV-1. The interaction between the viral envelope, which contains coreceptor-binding site, and the CCR5 on DCs mediates the cellular movement. HIV-1 enters between adjacent epithelial cells and DCs form junctions with epithelial cells, possibly driving their directional motility.

**IMPACT**

Our findings describe an active role of DCs in translocating HIV-1 across the intestinal epithelium. Remarkably, this phenomenon is induced only by viruses using CCR5 as coreceptor, which is of relevance considering that this is the viral phenotype preferentially transmitted *in vivo*.

This novel mechanism may be targeted for the design of new prevention strategies, as viral envelopes with the characteristic to induce DCs to sample luminal virions may be exploited in mucosal vaccination strategies to favour antigen presentation.

### Immunofluorescence markers and confocal microscopy

Cell nuclei were labelled with DAPI, DCs with anti-CD11c or anti-DC-SIGN, TJs with Occludin, ZO-1 and JAM, adherent junctions with E-Cadherin, colonic epithelial cells with anti-epithelial antigen (HEA) and colonic Mϕ with anti-CD68. Virions were visualized by GFP expression or alternatively stained with anti-p24 mAb or anti-gp120 2G12 mAb. As negative control, HIV-1-treated samples were stained with secondary antibody alone, whereas medium-treated samples with both primary and secondary antibodies. When indicated, cells were treated for 1.5 h with HIV-1^AD8GFP^ (100 ng of p24Ag) in presence or absence of ovalbumin Alexa Fluor647 conjugate (Invitrogen), and Lyso Tracker Red DND-99 (Invitrogen), then washed and fixed with 2% PFA to be analysed at CM.

Single channel images from *z*-series were collected from at least three representative fields with a Leica TCS SP5 AOBS confocal microscope (Leica Microsystems GmbH, Wetzlar Germany), then 2D free projection max images were obtained. Thereafter images were analysed with Volocity 5.0 (Improvision, Perkin–Elmer, Milano, Italy) and Adobe Photoshop CS (Adobe Systems, San Jose, CA) software.

DCs collected from the filter by centrifugation were rinsed twice in PBS, plated on poly(l) lysine-coated chamberslides for 2 h at 37°C, fixed in 1% PFA (10 min, RT) and processed for immunofluorescence. As control 1 × 10^6^ DCs were plated on poly(L) lysine–treated chamberslides and incubated with HIV-1 supernatant (10 ng of p24 Ag input) for 1.5 h. DCs were stained with anti-DC-SIGN-PE antibody, virus was visualized either by GFP expression or with anti-p24 mAb. Fully stained medium-treated samples and HIV-1-treated samples where the primary antibody was omitted were used as negative controls.

### Electron microscopy analysis

Specimens incubated in the presence of HIV-1 supernatant or negative control medium for 30 min, 90 min or 24 h, were processed as previously described (Foglieni et al, 2005) for inclusion in EmBed-812 resin. Briefly, the membrane was excised from the support, fixed with 2.5% glutaraldehyde in 0.1 M cacodylate buffer (pH 7.4, overnight, 4°C), rinsed in cacodylate buffer, stained with 1% tannic acid post-fixed with osmium tetroxide (15 min, RT). After dehydration at increasing concentrations of ethanol:H_2_O (40, 70, 90 and 100% ethanol, 5 min each) followed by propylene oxide (5 min, RT), infiltration in propylene oxide:resin (1:1 v:v, 60 min, RT) and thereafter in resin (overnight, RT), the filter resin blocks were allowed to polymerize (3–5 days, at 58°C). Thin sections on formvar–supported nickel grids were stained with uranyl acetate/lead citrate and analysed under TEM (LEO 912AB, Carl Zeiss SMT AG) equipped with a digital camera system. All the reagents and supplies for TEM were from Electron Microscopy Sciences (Fort Washington, PA, USA).

### DCs morphometry

The entity of DCs migration in Caco-2/DCs co-culture system was evaluated in representative experiments using specimens treated either with medium only, LPS, HIV-1^AD8^, HIV-1^pNL4.3^, HIV-1^YU2^, HIV-1^92UG023^, YU2 gp120 protein or CN54 gp140 protein. Caco-2 cells were stained for JAM and DCs for DC-SIGN and visualized by CM. To obtain comparable levels between different experiments, images obtained at two different levels along the *z*-axis, *i.e.* at a distance of 2.5 µm from the Caco-2 cells surface (apical level) and exactly in the middle of the cell height (medial level) respectively, were selected from each *z*-series. Three or four *z*-series from three different experiments (acquired with objective 40×) were analysed each for the apical and the medial level. The area occupied by DCs and their protrusions was measured with ImageJ and expressed as percentage compared to the total area of the *z*-series.

### Statistical analysis

Univariate repeated measures analysis (split-plot design) (SAS Software, release 9.2, SAS Institute inc.) and Bonferroni *t*-test of differences between means for the considered treatments were applied to evaluate the significance in DCs migration efficiency. Non-parametric Mann–Whitney test was performed for infection assays to assess statistical significance of data. *p*-values <0.05 indicate statistical significance.

## Author contributions

MC has performed most of the experiments; CF has contributed with the CM and TEM; MR provided the expertise for the Caco-2/DCs co-culture system; GS initiated the project and supervised the work. All contributed to the writing and correction of the manuscript.

## Notes

As a service to our authors and readers, this journal provides supporting information supplied by the authors. Such materials are peer reviewed and may be re-organized for online delivery, but are not copy-edited or typeset. Technical support issues arising from supporting information (other than missing files) should be addressed to the authors.
