# Supplementary material for: R5 HIV-1 envelope attracts dendritic cells to cross the human intestinal epithelium and sample luminal virions via engagement of the CCR5
Source: EMBO Mol Med. 2013 Apr 22;5(5):776–94. doi: 10.1002/emmm.201202232 (PMC3662319; doi:10.1002/emmm.201202232)
Supplement: Supplementary file 1 [file emmm0005-0776-sd1.pdf]

R5 HIV-1 envelope attracts dendritic cells to cross the human intestinal epithelium and sample luminal virions via engagement of the CCR5

Mariangela Cavarelli, Chiara Foglieni, Maria Rescigno and Gabriella Scarlatti

Corresponding author: Mariangela Cavarelli, San Raffaele Scientific Institute

Review timeline:

Submission date:	02 November 2012
Editorial Decision:	13 January 2013
Revision received:	21 February 2013
Accepted:	08 March 2013

Transaction Report:

(Note: With the exception of the correction of typographical or spelling errors that could be a source of ambiguity, letters and reports are not edited. The original formatting of letters and referee reports may not be reflected in this compilation.)

Editors: Natascha Bushati / Céline Carret

1st Editorial Decision 13 January 2013

Thank you for the submission of your manuscript to EMBO Molecular Medicine. I am sorry that it has taken so long to get back to you on your manuscript.

While reviewers 1 and 3 delivered their evaluations in a timely manner, we did not receive the other reviewers' input. As the evaluations from the first two reviewers are consistent, and a further delay cannot be justified, I have decided to proceed based on these evaluations.

If in the meanwhile we should receive the other reviews within the next 10 days and only if they raise significant caveats, these would need to be taken into consideration. We would not, however, ask you to comply with any further-reaching requests.

You will see that while both reviewers are generally supportive of your work and underline its considerable potential interest, they also raise a number of specific concerns that prevent us from considering publication at this time.

Importantly, reviewer 3 requires quantification and further analysis of the migration and adhesion of DCs to the apical membrane. This reviewer also suggests analyzing whether the HI virus remains intact in an intracellular compartment of DCs. In addition, reviewer 3 suggests to analyze the production of CCL20 upon in Caco2 cells upon viral stimulation.

Reviewer 1 requires clarifying a number of issues, including various controls and moving some of the supplemental data into the main body of the manuscript.

Finally, the reviewers have a number of important suggestions to improve interpretation of your

data, impact and readability of your work.

Should you be able to address the raised concerns with additional experiments where appropriate, we would be willing to consider a revised manuscript.

Please note that it is EMBO Molecular Medicine policy to allow a single round of revision in order to avoid the delayed publication of research findings. Consequently, acceptance or rejection of the manuscript will depend on the completeness of your responses included in the next version of the manuscript.

EMBO Molecular Medicine has a "scooping protection" policy, whereby similar findings that are published by others during review or revision are not a criterion for rejection. Should you decide to submit a revised version, I do ask that you get in touch after three months if you have not completed it, to update us on the status.

Please also contact us as soon as possible if similar work is published elsewhere. If other work is published we may not be able to extend the revision period beyond three months.

I look forward to receiving your revised manuscript.

***** Reviewer's comments *****

Referee #1 (Comments on Novelty/Model System):

This paper addresses an important issue in HIV biology, the initial interaction of the virus with target cells in the (intestinal) mucosa and is well done with clear figures and (mostly) good controls and much data - perhaps too much ins supplemental figures. The phenomena described are novel and two types of model are presented, one being native colonic explants and the other using less representative malignant colonic epithelial cells cells and monocyte derived DCs, and are mutually reinforcing. (MDDCs are quite closely related to lamina propria/dermal DCs.

Referee #1 (General Remarks):

The role of dendritic/Langerhan's cells as the first cells to be targeted by HIV in the genital stratified squamous epithelium is still not completely accepted. Therefore research on such initial interactions between DCs and HIV remains a topic of continuing interest. In this interesting paper the authors examined the transmission of HIV across the colonic intestinal epithelium in explant culture and in a model system of CaCo cells. The latter form a continuous epithelial monolayer on membranes of transwells with the application of monocyte derived dendritic cells to the under surface. Both were exposed on the apical surface to R5 and X4 strains of HIV. They show data indicating that the DCs extend dendrites through the intracellular junctions to the lumen after apical application of R5 but not with X4 HIV strains. Both R5 and X4 strains are transported through the epithelial layer via the intracellular or transcellular route. Both strains can then be transferred from DCs to PBMCs. Their data seems to show that persistence of this transfer is probably associated with persistent replication of (R5) HIV within the DCs

The experiments are mostly well performed and images are mostly clear and well labelled, as are the legends. However some of the experiments require extra controls and others clarification. There is extensive supplemental data and some of this should be in the main body of the manuscript.

Such HIV stimulated DC process extension or DC migration to the surface has not been reported in stratified squamous epithelium (cervix/foreskin) so it may be a particular property of certain epithelia. Therefore can they further justify the relevance of the model to post-natal transmission from breast milk by using colonic epithelial cells? Similarly as most of the transmission via anal intercourse occurs presumably either above or below the dentate line (i.e. through rectal columnar epithelium above or stratified squamous epithelial below with a transition in between) how similar are their colonic epithelial explants to the site of transmission in rectal epithelium?

Specific Questions

Figure 1: Do whole whole DCs migrate between the epithelium or just their processes? Can DC nuclei be stained in Figs 1-4?
The results for J6363 and 3V strains of HIV should also be included in the bar graphs in panels G and J instead of in supplemental data (S1).

Figure 2A: An electron micrograph of the CaCo cells on filters alone should be shown as a control and the apparent presence of DCs in the pores and above the filter membrane is justified ie how are these cells differentiated ultrastructurally from CaCo cells? The supplemental figure S4, which should be in the main body of the MS, only partly satisfies these concerns. This is especially important as on page 6 DCs are reported to establish TJ like structures with CaCo cells! Where are they in the figure and would they not impede migration?

Figure 4,
panel B. The HIV particles embedded in mucus should be shown with arrows.
Panel D: The inset is not convincing as a demonstration of dendritic cell extensions penetrating the epithelial cell monolayer.
Panels E and F: some of the green particles have bulk and shape so they cannot be individual viral particles. Are they viral aggregates or fluorescent artefact? Where is the GFP label found in the virion and have they performed assessments of inoculum virus alone on slides?
General: Is most of the HIV conveyed across the CaCo or colonic epithelium by transcytosis, via intercellular junctions, does this differ in kinetics or by R5/X4 type?

Figure 5: The experiments with the V3 loop deletion mutants are important controls and figure S7 should be shown in the main body of the MS.

Figure 6: DCs were incubated with Maroviroc and CCL5 before attaching to the transwell membrane. Did this affect the numbers adhering to the filter? Why not add the reagents after adherence? Does CCL5 alone cause the process extension induce by HIV

They suggest R5 virions alone may be chemotactic for DCs or their processes. Is there any precedent for this? HIV has been shown to induce DC maturation and migration, both after vesicular uptake and CCR5 mediated fusion infection (Wilflingseder D et al 2004 , Harman A et al 2006, Anand AR et al 2009). These papers should be cited and the relationship to their hypothesis discussed eg are they suggesting CCR5 signalling is responsible for the migration?

The transfer of X4 HIV from MDDCs to CD4 lymphocytes is postulated to occur predominantly via vesicular uptake rather than de novo infection. Is this consistent with their finding in Figure 9? In discussion they should refer to these two different pathways and cite relevant literature (eg Turville S et al 2004)
In figure 10 they show only DC processes extending between the epithelial cells. Do whole cells also migrate as suggested in figures 1-4 and S4?

Referee #3 (Comments on Novelty/Model System):

This paper presents convincing evidence on the mechanisms whereby dendritic cells are are ble to migrate across a tight monolayer of epithelial cells in response to R5 HIV and R5 gp140, but not X4 virus and glycoprotein. The paper is original and novel and merits publication in EMBO Molecular Medicine. The strength of the paper is the validation of an in vitro co-culture system by ex-vivo studies using human colonic explants inserted into modified Ussing chambers. The model is appropriate to address the role of HIV in DC recruitment and dissecting which determinant on the virus are able to trigger migration. There are some points which need clarification and which are spelled out in the remarks send to the authors.
Whether such an approach will contribute to the design of novel preventive strategies is questionable, but it clearly indicates that microbicide and vaccine approaches will have to take into account the results of this study. In conclusion I recommend that the paper be published after

revisions indicated below

Referee #3 (General Remarks):

This paper present convincing evidence on the mechanisms whereby dendritic cells are able to migrate across a tight monolayer of epithelial cells in response to R5 HIV and R5 gp140, but not X4 virus and glycoprotein. The study is timely and medically relevant and merits publication in EMBO Molecular Medicine. Prior publication, however, some points have to be addressed:
1/. The authors claim that DCs migrate through a tight monolayer. They show that DCs reach the apical membrane where they remain attached both in the co-culture system and the human colonic explants, but fail to provide information about the number of DCs that are released in the apical medium. The nature of the adhesion of DCs to the apical is not analyzed. Which cell adhesion molecules mediate attachment should be analyzed. Is the attachment reversible. This is important if the cells have to transmit HIV by migrating back to the lamina propria and eventually in the draining lymph node. Similarly, the number of HIV loaded DCs present in the basal chamber is not analyzed. Such data should be presented in a table.
2/ The authors in Fig 9 mention that viral production in DCs cultured alone in the absence of PBMCs is low. This would indicate that HIV does not infect DCs. It has recently been reported that DCs are able to take up antigens in a non degradative compartment and release them from a late endosome compartmeent upon interaction with target cells (Le Roux et al. 2012. Blood 119:95-105). The authors have all the tools required to analyze whether the virus remains intact in an intracellular compartment . If this is the case, it has implication on vaccine strategies. Indeed cytotoxic T cells would be inefficient in controlling the early stages of mucosal HIV infection.
3/. In order to identify which cytokines/chemokines released by Caco2 cells upon viral stimulation could mediate recruitment of DCs, the authors measured the 3 chemokines CCL3,4 and 5. Their production did not correlate with R5 HIV exposure. CCL20 known to be produced by epithelial cells has not been analyzed. Such a measurement should be provided. In addition it is also possible that DCs themselves in response to R5 virus stimulation may participate in the process by releasing CCl20. It has been reported that DCs in response to TLR agonists produce CCL20 among other cytokines and chemokines (Jensen SS and Gad M.2010. J. Inflamm (London)7:37).
4/ Finally the legends of figures could be improved. Presently it is difficult to find out which cultures are incubated with which R5 or X4 viruses. It would help if the authors add this information on the name of the different viral strains used in the study.

1st Revision - authors' response 21 February 2013

Referee #1 (Comments on Novelty/Model System):

This paper addresses an important issue in HIV biology, the initial interaction of the virus with target cells in the (intestinal) mucosa and is well done with clear figures and (mostly) good controls and much data - perhaps too much ins supplemental figures. The phenomena described are novel and two types of model are presented, one being native colonic explants and the other using less representative malignant colonic epithelial cells and monocyte derived DCs, and are mutually reinforcing. (MDDCs are quite closely related to lamina propria/dermal DCs.

Referee #1 (General Remarks):

The role of dendritic/Langerhan's cells as the first cells to be targeted by HIV in the genital stratified squamous epithelium is still not completely accepted. Therefore research on such initial interactions between DCs and HIV remains a topic of continuing interest. In this interesting paper the authors examined the transmission of HIV across the colonic intestinal epithelium in explant culture and in a model system of CaCo cells. The latter form a continuous epithelial monolayer on membranes of transwells with the application of monocyte derived dendritic cells to the under surface. Both were exposed on the apical surface to R5 and X4 strains of HIV. They show data indicating that the DCs extend dendrites through the intracellular junctions to the lumen after apical application of R5 but not with X4 HIV strains. Both R5 and X4 strains are transported through the epithelial layer via the intracellular or transcellular route. Both strains can then be

transferred from DCs to PBMCs. Their data seems to show that persistence of this transfer is probably associated with persistent replication of (R5) HIV within the DCs
The experiments are mostly well performed and images are mostly clear and well labeled, as are the legends. However some of the experiments require extra controls and other clarifications. There is extensive supplemental data and some of this should be in the main body of the manuscript.

Reply: we thank the reviewer for his/her positive comments

Such HIV stimulated DC process extension or DC migration to the surface has not been reported in stratified squamous epithelium (cervix/foreskin) so it may be a particular property of certain epithelia. Therefore can they further justify the relevance of the model to post-natal transmission from breast milk by using colonic epithelial cells? Similarly as most of the transmission via anal intercourse occurs presumably either above or below the dentate line (i.e. through rectal columnar epithelium above or stratified squamous epithelial below with a transition in between) how similar are their colonic epithelial explants to the site of transmission in rectal epithelium?

Reply: We agree with the referee that the mechanism of HIV transmission may vary according to the different anatomical sites, i.e. the gastrointestinal mucosa and the vaginal mucosa. In the future it will be extremely interesting to extend our study to other mucosal tissues. Although a specific DC migration across the pluri-stratified epithelium of the cervix or foreskin has never been reported, it is also true that Langherans cells (LC) located inside such pluri-stratified epithelia have been repeatedly described as one of the first cells sampling HIV (Hladik 2007, Hu 2000, Shen 2010, Spira 1996). Moreover, in a recent study it has been shown that epidermal LC were located in the apical surface of the inner foreskin when exposed to HIV (Zhou Z. 2011). We have mentioned this in the introduction, third paragraph, page 4 and in the discussion at page 16 of the revised manuscript. We added a sentence in the discussion (first paragraph, page 13) to underline the need to explore the described mechanisms also in other tissues.

We agree with the referee that transmission via anal intercourse occurs presumably either above or below the dentate line. However, it has been demonstrated that semen can travel at least 60cm up the colon, which means that a large surface area of those tissues is potentially exposed to semen during unprotected sex (Louissaint NA, JAIDS 2012). Analogously in the setting of mother to child transmission HIV is not inactivated in the stomach due to the considerably higher pH of the infant stomach compared to that of the adult (Mason S , Arch Dis Child 1962), and thus the virus can reach the intestine. This allows us to consider the colonic tissues we used in this manuscript to be a relevant model for a proof of concept showing that DCs can migrate to capture the virus.

Specific Questions

Figure 1: Do whole DCs migrate between the epithelium or just their processes? Can DC nuclei be stained in Figs 1-4?
The results for J6363 and 3V strains of HIV should also be included in the bar graphs in panels G and J instead of in supplemental data (S1).

Reply: We detected both processes as well as whole DCs in the epithelial monolayer, the latter ones being more frequent at longer incubation times as demonstrated by time-course experiments described in the Results section, first chapter, page 6, and in Fig S3.
We regret we cannot show DC nuclei in Fig 1 because the cells were not labeled with DAPI at the time of the experiment. However we labeled DC nuclei in several other figures, including Fig 2,4,5,6,7 and S3. We believe that in this way we show in the manuscript different staining combinations as to give to the reader a wide array of different approaches.
In accordance with the referee's comment we have added the quantification of the DCs migration in response to HIV J6363 and IIIB as bar graph in Figure 1 G and J of the revised manuscript.

Figure 2A: An electron micrograph of the CaCo cells on filters alone should be shown as a control and the apparent presence of DCs in the pores and above the filter membrane is justified i.e. how are these cells differentiated ultra structurally from CaCo cells? The supplemental figure S4, which should be in the main body of the MS, only partly satisfies these concerns. This is especially

important as on page 6 DCs are reported to establish TJ like structures with CaCo cells! Where are they in the figure and would they not impede migration?

Reply: In accordance with the referee's request we are now showing Caco-2 cells monolayer as negative control in Fig S5 of the revised manuscript.
Purpose of the TEM analysis was to confirm the data obtained at CM that HIV-1 treatment and DCs migration would not affect the continuity of the epithelial cells monolayer by disrupting junctional connections among Caco-2 cells. We observed that Caco-2 cells have columnar shape, big nucleus, brush border, huge amount of vesicles in the apical regions and a complete Junctional system. In this setting, DCs have irregular shape, frequently with pseudopodes interposed in between epithelial cells, and small nuclei. We have described these features in figure legend of Figure2. We are aware that this ultrastructural observation does not represent a definitive proof of the nature of the cells, however we feel confident to say that it supports the data obtained at CM.

Figure S4 was moved to the main body of the manuscript as suggested by the referee. To avoid redundancy we have fused figure 2 with figure S4. The new figure is numbered as Figure 2 in the revised manuscript.

The referee is asking if the junctions between DC and Caco-2 cells would not impede the migration of DCs. Rescigno, co-author of this manuscript, has published (Nat Immunol 2001) that the TJs formation, indeed, allows the DCs to open up the TJ and migrate across the intestinal epithelium when stimulated with bacteria. To underline and better specify this aspect we added a sentence in the Results section first chapter, page 6, which states “This is in line with our previous results in mice that DCs migrating between epithelial cells following stimulation with bacteria preserve the epithelial barrier due to TJ-like structures between DCs and Caco-2 cells”. We also introduced a Figure (S4) with the expression of the TJ proteins (occluding, ZO-1 and Jam) on the DC used in our experimental setting as determined by cytofluorimetric analysis.

Figure 4,
panel B. The HIV particles embedded in mucus should be shown with arrows.

Reply: We have added arrows to Figure 4 (Fig 5 in the revised manuscript) to help discriminate virions entrapped in the mucus.

Panel D: The inset is not convincing as a demonstration of dendritic cell extensions penetrating the epithelial cell monolayer.

Reply: In accordance with the referee’s concern, in the revised manuscript we show a new figure (Figure 5 C and D) which clearly shows a DC penetrating the epithelium.

Panels E and F: some of the green particles have bulk and shape so they cannot be individual viral particles. Are they viral aggregates or fluorescent artifact? Where is the GFP label found in the virion and have they performed assessments of inoculum virus alone on slides?

Reply: We agree with the referee that the description of the GFP-labeled virions was insufficient in the Material section. For completeness, we have added in the Methods section (page18) a description of how GFP-tagged HIV was produced. The sentences phrases now: “GFP-Vpr-labeled HIV-1AD8, designated as HIV-1AD8GFP throughout the manuscript, was produced by transfection of 293T cells with the proviral construct pAD8 and the plasmid pGFPC3 (kind donation of Dr. Paxton) containing the entire Vpr coding region fused to the carboxy-terminus of eGFP (GFP-Vpr) as previously described (McDonald et al, 2002).” Thus, the GFP protein is fused to the Vpr protein and is incorporated into the core of the virion.

It is conceivable that many of the green particles observed in Panels E and F may be viral aggregates as suggested by the referee. To underline this aspect, we have now introduced the following sentence in the revised manuscript (page 8) “Some of the particles presented bulk and shapes, suggesting that they may be viral aggregates more than individual viral particles (Fig 5E,F)”. Moreover, to further show that the green particles visualized at confocal microscopy on the DCs were HIV and not artifacts, we added flow cytometric analysis which shows the percentage of HIV-1-GFP+ DCs. We show this result in Fig S6 of the revised manuscript and added a sentence in the

Results section (page 8) which states: "GFP+ virions were also detected on DCs after detachment from the filter (Fig S6 of Supporting Information) by both CM and flow cytometry".

General: Is most of the HIV conveyed across the CaCo or colonic epithelium by transcytosis, via intercellular junctions, does this differ in kinetics or by R5/X4 type?

Reply: Accordingly with the referee's observation in the revised manuscript we show in Table S1 that no difference was observed in kinetic of transcytosis between R5 and X4 viruses. We changed also the text in the results section (page 11) to specify this. The text is now: "The kinetic experiments performed incubating R5 HIV-1^{AD8} and X4 HIV-$1^{pNL4.3}$ on the apical side of Caco-2 cells at regular time intervals between 30 min and 4.5 h confirmed previous published data (Bomsel, 1997), that transcytosis is time-dependent and reaches a peak after approximately 3 h independently of the viral phenotype (Table S1 of Supporting Information), being, thus, a delayed process and not selective for R5 viruses compared to DCs migration."
Indeed, we observed the passage of virus via intercellular route less frequently compared to the transcytotic pathway. However, we regret that the experimental model we use does not allow to perform quantitative analysis of the virus that is conveyed via intercellular junctions. We prefer not to comment on this aspect.

Figure 5: The experiments with the V3 loop deletion mutants are important controls and figure S7 should be shown in the main body of the MS.

Reply: In accordance with the referee's request we have moved the figure S7 in the main body of the manuscript. The new figure is numbered as Fig 7 in the revised manuscript.

Figure 6: DCs were incubated with Maroviroc and CCL5 before attaching to the transwell membrane. Did this affect the numbers adhering to the filter? Why not add the reagents after adherence? Does CCL5 alone cause the process extension induce by HIV.

Reply: We believe that there has been a misunderstanding. Indeed the experiments mentioned by the referee were performed incubating the reagents before or during the attachment of the DCs to the filter (as stated in result section, last paragraph of chapter "DC migration across the epithelial monolayer", page 9). The experiments incubating before attachment were only performed to exclude an effect due to a reverse chemotactic gradient induced by the chemokines. Whereas addition of the chemokines to the DCs after adhesion to the filter would have changed completely the experimental setting compared to all other experiments. Furthermore, we would not be able to control appropriately the degree of down-modulation of CCR5 after 2-4h of chemokine treatment.

The concern of the referee that the treatment may affect the numbers of DCs adhering to the filter, was already appointed in the original manuscript in the Methods section, page 19 . The text reads "When indicated, DCs were treated with the CCR5 ligand/inhibitor CCL5 (200 ng/ml) or Maraviroc (100 ng/ml) for 2 or 4 h. The different treatments did not impact on the attachment of DCs to the filter as verified at CM". We carefully checked in all the experiments that DCs were homogenously distributed along the filter, and excluded the experiment when such condition was not satisfied. For the referee's information only, we include here a representative experiment showing that filters treated or not with CCL5 displayed a similar amount of attached DCs compared to controls. If the referee considers this information vital we can include this picture as Supplementary information.

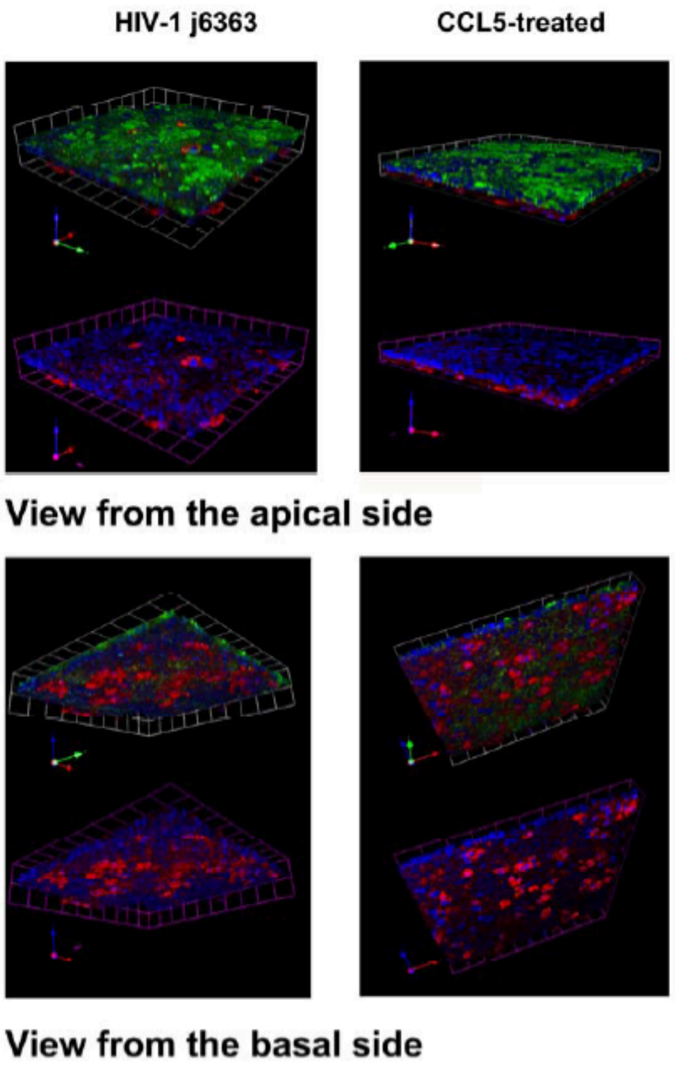
HIV-1 j6363
CCL5-treated
View from the apical side
View from the basal side

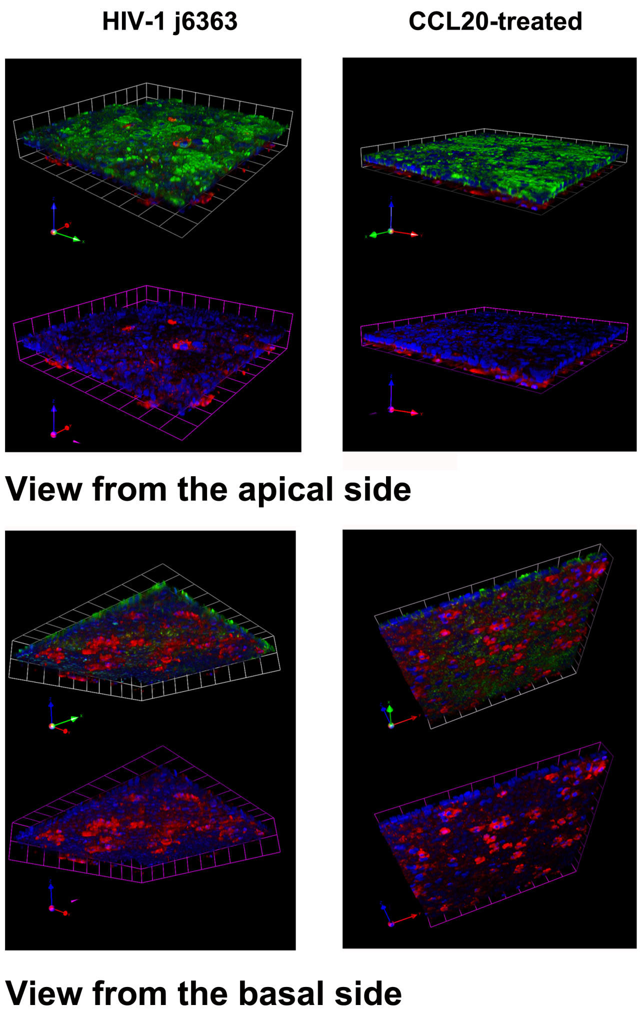

For the referee we know that CCL5 alone can induce DCs migration.

They suggest R5 virions alone may be chemotactic for DCs or their processes. Is there any precedent for this? HIV has been shown to induce DC maturation and migration, both after vesicular uptake and CCR5 mediated fusion infection (Wilflingseder D et al 2004, Harman A et al 2006, Anand AR et al 2009). These papers should be cited and the relationship to their hypothesis discussed e.g. are they suggesting CCR5 signalling is responsible for the migration?

Reply: It has been shown that R5 HIV-1 is chemotactic for DCs acting through CCR5 binding (Lin CL, J Exp Med 2000), and we cited the paper by Lin et al in the Discussion at page 14.
We thank the referee for the suggestion to discuss our data in the context of relevant literature and better specify that CCR5 signaling may be responsible for the DC migration. Thus, we have added the following paragraph in the "Discussion" section: "It was described that HIV-1 gp120 binding to CCR5 activates a signaling cascade leading to phosphorylation of the nonreceptor tyrosine kinase Pyk2, and downstream activation of p38MAPK, which ultimately leads to DC migration (Anand et al, 2009; Harman et al, 2006; Wilflingseder et al, 2004). Thus, it is conceivable that also in our model, signaling through CCR5 may be required to induce DC migration across the epithelium".

The transfer of X4 HIV from MDDCs to CD4 lymphocytes is postulated to occur predominantly via vesicular uptake rather than de novo infection. Is this consistent with their finding in Figure 9? In discussion they should refer to these two different pathways and cite relevant literature (e.g. Turville S et al 2004)

© EMBO

Reply: We thank the referee for the comment. We believe that our results are consistent with those by Turville et al. Indeed, the DCs used in our model express low levels of CXCR4 (below 2%), which thus, would not favor cis-infection by X4 virus, and high levels of DCSIGN (about 90%), which is mainly involved in the vesicular uptake and trans-infection pathway. We have discussed the two pathway of infection and cited the relevant literature as suggested by the referee in the Discussion at page 16-17.

In figure 10 they show only DC processes extending between the epithelial cells. Do whole cells also migrate as suggested in figures 1-4 and S4?
Reply: We have revised the figure (numbered as Figure 12 in the revised manuscript) to show also whole DC migrating across the epithelium, as also seen in our experimental set-up with the Caco-2/DCs and the explant cultures.

Referee #3 (Comments on Novelty/Model System):
This paper presents convincing evidence on the mechanisms whereby dendritic cells are able to migrate across a tight monolayer of epithelial cells in response to R5 HIV and R5 gp140, but not X4 virus and glycoprotein. The paper is original and novel and merits publication in EMBO Molecular Medicine. The strength of the paper is the validation of an in vitro co-culture system by ex-vivo studies using human colonic explants inserted into modified Ussing chambers. The model is appropriate to address the role of HIV in DC recruitment and dissecting which determinants on the virus are able to trigger migration. There are some points which need clarification and which are spelled out in the remarks send to the authors.
Whether such an approach will contribute to the design of novel preventive strategies is questionable, but it clearly indicates that microbicide and vaccine approaches will have to take into account the results of this study. In conclusion I recommend that the paper be published after revisions indicated below

Referee #3 (General Remarks):
This paper present convincing evidence on the mechanisms whereby dendritic cells are able to migrate across a tight monolayer of epithelial cells in response to R5 HIV and R5 gp140, but not X4 virus and glycoprotein. The study is timely and medically relevant and merits publication in EMBO Molecular Medicine. Prior publication, however, some points have to be addressed:

Reply: We thank the reviewer for his/her positive comments on our paper.

1/. The authors claim that DCs migrate through a tight monolayer. They show that DCs reach the apical membrane where they remain attached both in the co-culture system and the human colonic explants, but fail to provide information about the number of DCs that are released in the apical medium. The nature of the adhesion of DCs to the apical is not analyzed. Which cell adhesion molecules mediate attachment should be analyzed. Is the attachment reversible? This is important if the cells have to transmit HIV by migrating back to the lamina propria and eventually in the draining lymph node. Similarly, the number of HIV loaded DCs present in the basal chamber is not analyzed. Such data should be presented in a table.

Reply: We thank the referee for the interesting observations. In accordance with the referee's request we have analyzed the number of DCs that are released in the apical medium. The results are presented in the “Results” section of the revised manuscript (page 6). The text reads: “migrated DCs were not massively released into the apical chamber. Indeed, after 4 h of incubation with HIV-1AD8 only a limited number of DCs (4 ± 5 cells) were detected in the apical chamber compared to none in the negative control with medium. DCs expressed the TJ proteins Occludin and ZO-1, the Junction-Adhesion-Molecule (JAM) and the β2 integrin LFA-1 (Fig S4 of Supporting Information), which may favor retainment of DCs attached to the epithelium”.

Although we find it to be an important and compelling issue, in this work we did not focus on adhesion molecules mediating the apical interaction between intestinal epithelial cells and DCs. We hope the referee agrees with us that the study of the adhesion molecules involved in retaining DCs at the apical side of the epithelium is a study *per se.*

We thank the referee for the comment on the reversibility of migration of DCs. This is a very important point to further support the relevance of our results in infection, transmission and antigen presentation. We have added some additional experiments (Fig. 3) to show that the migration is completely reversible when a chemotactic agent is added to the basal side. In the revised manuscript we added the experimental conditions in Material and methods (page 20), and a paragraph in the "Results" section (page 6-7), that states: "We sought to investigate whether DCs, once migrated into the epithelial monolayer, were able to return to the basal chamber after an appropriate stimulus, such as the CX3CR1-binding chemokine fractalkine, known to induce DC migration (Dichmann et al, 2001). As shown in Fig 3, incubation with fractalkine completely reversed the migration of DCs to the basal level. Thus, DCs can shuttle across the epithelial barrier and may thus, vehicle captured virus to replication competent cells in the mucosa or lymph nodes".
About the comment on the number of HIV loaded DCs present in the basal chamber, we regret to say that such type of analysis cannot be accurately performed due to two reasons. 1) As we stated in the Results section (page 8), the presence of virions on non-migrated DCs was a rare event, which does not allow to perform a reliable quantitative analysis. We have reported the observation of this sporadic event only for completeness of information for the reader of the manuscript. 2) As DCs are homogenously distributed along the basal side of the Caco-2 cells and closely interact with each other, it is impossible to distinguish the limits of the single cells with confocal microscopy, and consequently it is impossible to count precisely the number of DCs harboring HIV virions. We do not feel confident to perform such analysis and hope the referee agrees with us.

2/ The authors in Fig 9 mention that viral production in DCs cultured alone in the absence of PBMCs is low. This would indicate that HIV does not infect DCs. It has recently been reported that DCs are able to take up antigens in a non degradative compartment and release them from a late endosome compartment upon interaction with target cells (Le Roux et al. 2012. Blood 119:95-105). The authors have all the tools required to analyze whether the virus remains intact in an intracellular compartment. If this is the case, it has implication on vaccine strategies. Indeed cytotoxic T cells would be inefficient in controlling the early stages of mucosal HIV infection.

Reply: In accordance with the referee's request we have added some experiments aimed at understanding whether the virus remained intact or not in intracellular compartments. We used GFP-tagged HIV to trace the virus travel through the DCs and fluorescent ovalbumin as to identify the retention compartment in the cell's cytoplasm. We also performed lysosome's staining as to understand whether the virus was redirected to a degradative compartment. We have added a figure (S10 of Supporting information), and the following paragraph in the "Results" section of the revised manuscript (page12): "We further traced the fate of the virus in the DCs by concomitant incubation with fluorescent ovalbumin and labeling with Lyso Tracker to discern the localization in acidic lysosomes. After 1,5 h of incubation with HIV-1AD8GFP about 60% of DCs harbored the virus, which was evenly distributed at the level of the cell membrane (Fig S10 of Supporting Information). Thereafter, at 30 minutes, most of the virus was within the cells but did not co-localize with neither ovalbumin-positive vesicles nor lysosomes. At 6 h viruses detected in less than 10% of cells, had accumulated in a perinuclear area. After 24 hours no virus was detected, which was consistent with a previous report (Yu et al, 2008). These results taken together with the recovery of virus by addition of PBMCs to DCs after 4 days in culture, suggest that the virus was also integrated in DCs". These results are discussed at page 16 of the revised manuscript.
We discuss these results in the Discussion section (page 17) of the revised manuscript.

3/. In order to identify which cytokines/chemokines released by Caco2 cells upon viral stimulation could mediate recruitment of DCs, the authors measured the 3 chemokines CCL3,4 and 5. Their production did not correlate with R5 HIV exposure. CCL20 known to be produced by epithelial cells has not been analyzed. Such a measurement should be provided. In addition it is also possible that DCs themselves in response to R5 virus stimulation may participate in the process by releasing CCl20. It has been reported that DCs in response to TLR agonists produce CCL20 among other cytokines and chemokines (Jensen SS and Gad M.2010. J. Inflamm (London)7:37).

Reply: We thank the referee for this comment. We have analyzed the possible release of CCL20 by Caco-2 cells and Caco-2/DC cultures, both apically and basally, after 1,5h and 24h of culture. CCL20 was found to be apically released by both Caco-2 cells and Caco-2/DC cultures after 24h of

stimulation, however with no difference between medium-treated and HIV-treated cultures. The results are shown at page 10 of the revised manuscript, and the methods section changed to include also CCL20 ELISA determination.

4/ Finally the legends of figures could be improved. Presently it is difficult to find out which cultures are incubated with which R5 or X4 viruses. It would help if the authors add this information on the name of the different viral strains used in the study.

Reply: We have added the information about the coreceptor usage of the virus used for each culture in all figure legends.

Accepted 08 March 2013

Please find enclosed the final report on your manuscript. We are pleased to inform you that your manuscript is accepted for publication and is being sent to our publisher to be included in the next available issue of EMBO Molecular Medicine.

***** Reviewer's comments *****

Referee #3 (General Remarks):

The authors have answered the questions raised by us and the paper can now be accepted for publication.
