# Supplementary material for: R5 HIV-1 envelope attracts dendritic cells to cross the human intestinal epithelium and sample luminal virions via engagement of the CCR5
Source: EMBO Mol Med. 2013 Apr 22;5(5):776–94. doi: 10.1002/emmm.201202232 (PMC3662319; doi:10.1002/emmm.201202232)
Supplement: Supplementary file 2 [file emmm0005-0776-sd2.pdf]

R5 HIV-1 envelope attracts dendritic cells to cross the human intestinal epithelium and sample luminal virions via engagement of the CCR5

Mariangela Cavarelli [1], Chiara Foglieni [1], Maria Rescigno [2], Gabriella Scarlatti [1]

SUPPORT INFORMATION

TABLE OF CONTENTS:

1 Supplemental Figures

S1: HIV-1 of R5 but not X4 phenotype induces DCs to migrate through a monolayer of epithelial cells

S2: DCs with comparable levels of CCR5 and CXCR4 migrate across the epithelium in response to R5 but not X4 HIV-1

S3: Kinetics of DCs migration across a Caco-2 monolayer following HIV-1 incubation

S4: DCs express tight junction proteins and adhesion molecules

S5: TEM micrograph of Caco-2 monolayer on transwell filter

S6: Virions are closely associated to DCs

S7: DCs migration across the epithelial monolayer in response to HIV-1 timeric gp140 protein is dose-dependent

S8: Down-regulation of CCR5 receptor on DCs treated with CCL5

S9: HIV-1 exposure does not alter junctional protein expression and permeability of the Caco-2 monolayer

S10: HIV-1 is rapidly internalized by DCs and is not redirected to an acidic lysosomal compartment

2 Supplemental Table

Table S1: Kinetics of HIV-1 transcytosis across Caco-2 cells.

SUPPLEMENTAL FIGURES

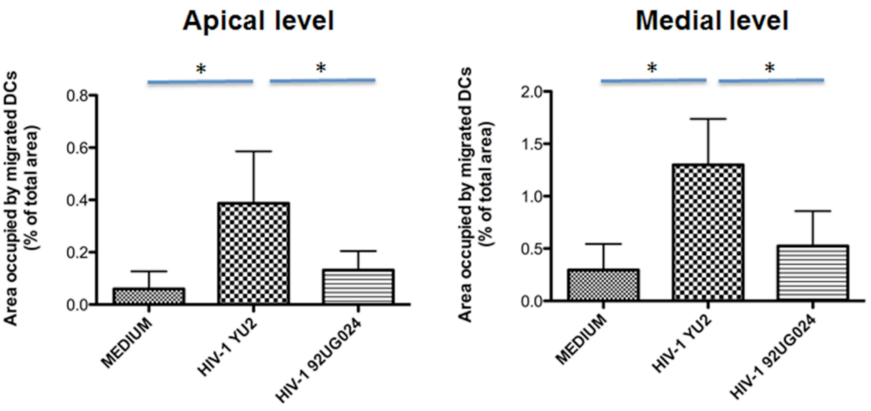

Fig S1 HIV-1 of R5 but not X4 phenotype induces DCs to migrate through a monolayer of epithelial cells.

Caco-2 cells were grown on transwell filter to form a confluent monolayer then DCs were let to adhere to the bottom of the filter. Cell-free HIV-1 of R5 (HIV-1^{YU2}) or X4 (HIV-1 ^{92UG024}) phenotype, or medium (DMEM 10% FCS) were incubated on the apical side of the Caco-2 monolayer for 1.5 h. Quantitative analysis of DCs migration across the Caco-2 cell monolayer at the apical (**A**) and medial (**B**) level of the cell layer is shown. Results are expressed as percentage of area occupied by DCs compared to that of the whole field. Bars represent mean ± SD of three or four fields of three different experiments. Statistic analysis was performed as described in Methods. * $p<0.05$.

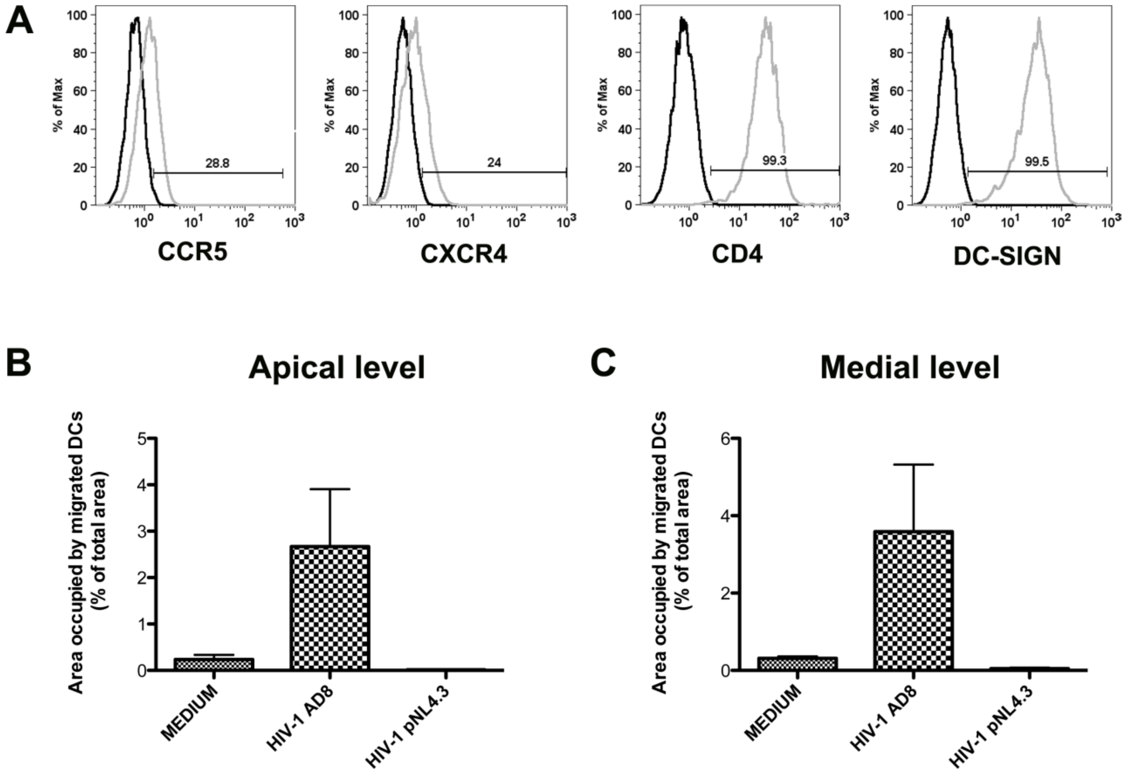

Fig S2. DCs with comparable levels of CCR5 and CXCR4 migrate across the epithelium in response to R5 but not X4 HIV-1.

(**A**) Cytofluorimetric analysis of HIV-1 receptors and coreceptors on DCs (treated with "colon-conditioned media" to up-regulate CXCR4 expression, as described in Materials and Methods section) show comparable levels of CCR5 and CXCR4 expression. Cells were labeled with mouse anti-human CCR5-FITC (clone 2D7), mouse anti-human CXCR4-PE (clone 12G5), mouse anti-human CD4-APC-Cy7 (clone RPA-T4) and mouse anti-human DC-SIGN-PE (clone 120507) . The black histogram is the negative control.

(B) and **(C)** Caco-2 cells were grown on transwell filter to form a confluent monolayer then DCs, which express comparable levels of CCR5 and CXCR4, were let to adhere to the bottom of the filter. Cell-free R5 HIV-1^{AD8}, X4 HIV-1$^{pNL4.3}$ or medium (DMEM 10% FCS) were incubated on the apical side of the Caco-2 monolayer for 1.5 h. Filters were processed for CM. Quantitative analysis

of DCs migration across the Caco-2 cell monolayer at the apical (**B**) and medial (**C**) level of the Caco-2 cell layer is shown. Results are expressed as percentage of area occupied by DCs compared to that of the whole field. Bars represent mean ± SD of three fields.

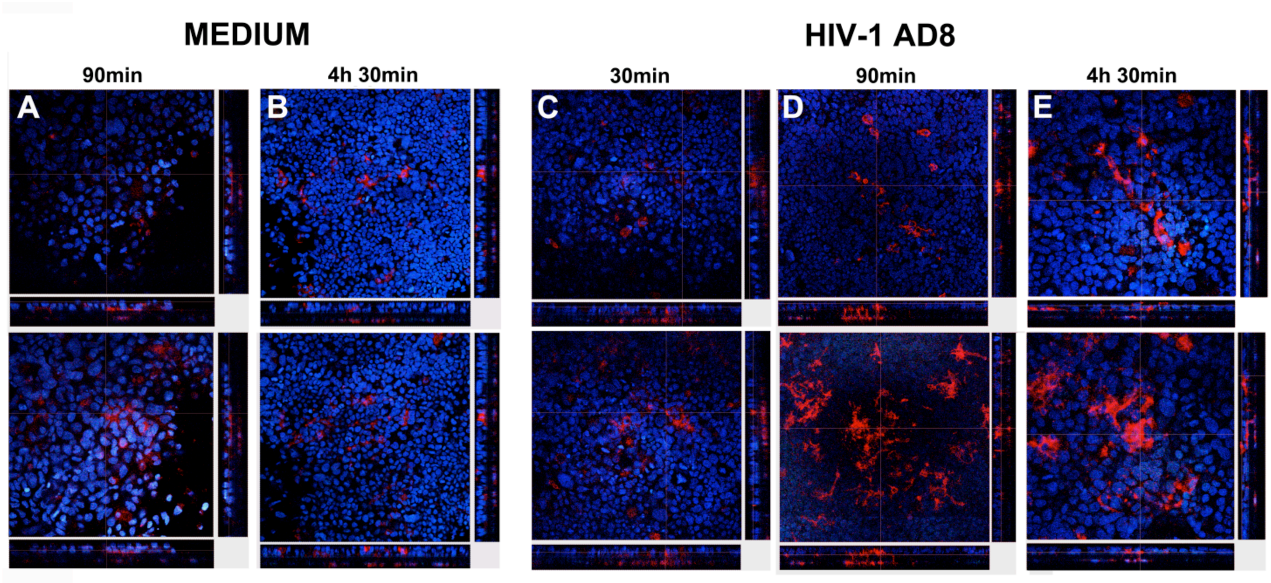

Fig S3 Kinetics of DCs migration across a Caco-2 monolayer following HIV-1 incubation.

Migration of DCs increased with time in the presence of virus. The Caco-2/DCs co-culture system was apically incubated with medium (**A** and **B**) or R5 HIV-1^{AD8} (20 ng of p24 Ag) (**C**-**E**) for 30 min, 90 min or 4.5 h. Cross sectional images from confocal microscopy z-series of representative fields from the Caco-2/DCs co-culture stained with DAPI nuclear dye (all cells; blue) and mouse anti-human DC-SIGN-PE (DCs; red) are shown (scale bar indicates the magnification). For each sample two planes along the z-axis are shown, one close to the apical side of the Caco-2 cells (upper panels), the other in the middle of the Caco-2 cells monolayer (lower panels). Results are of one representative experiment out of three.

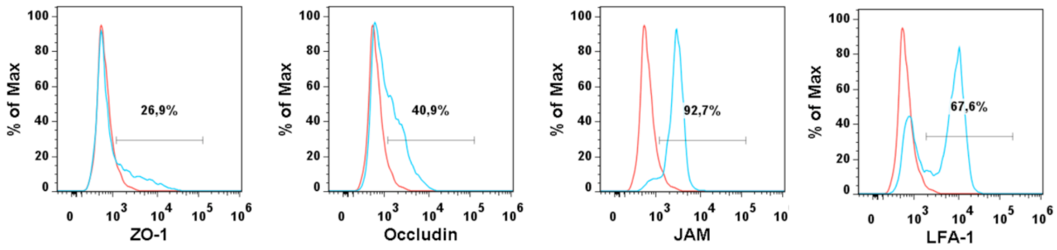

Fig S4 DCs express tight junction proteins and adhesion molecules.

Dendritic cells were stained with rabbit-anti-human ZO-1 + goat-anti-rabbit Alexa Fluor 488, rabbit-anti-human-occludin + goat-anti-rabbit Alexa Fluor 488, mouse-anti-human Junction-Adhesion-Molecule (JAM) + goat-anti-mouse FITC and mouse-anti-human LFA-1 + goat-anti-mouse FITC and analyzed by flow cytometry. Cells stained with secondary antibody alone were used as negative control.

Results are of one representative experiment out of three.

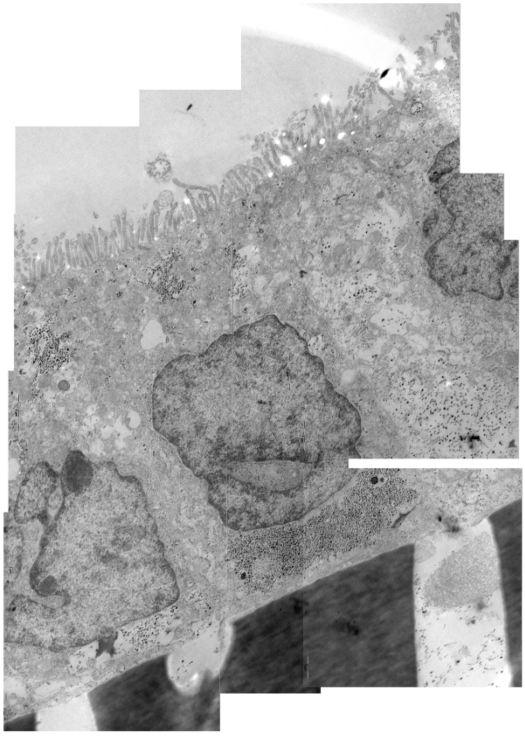

Fig S5 TEM micrograph of Caco-2 monolayer on transwell filter.

Shows a regular structure of the monolayer.

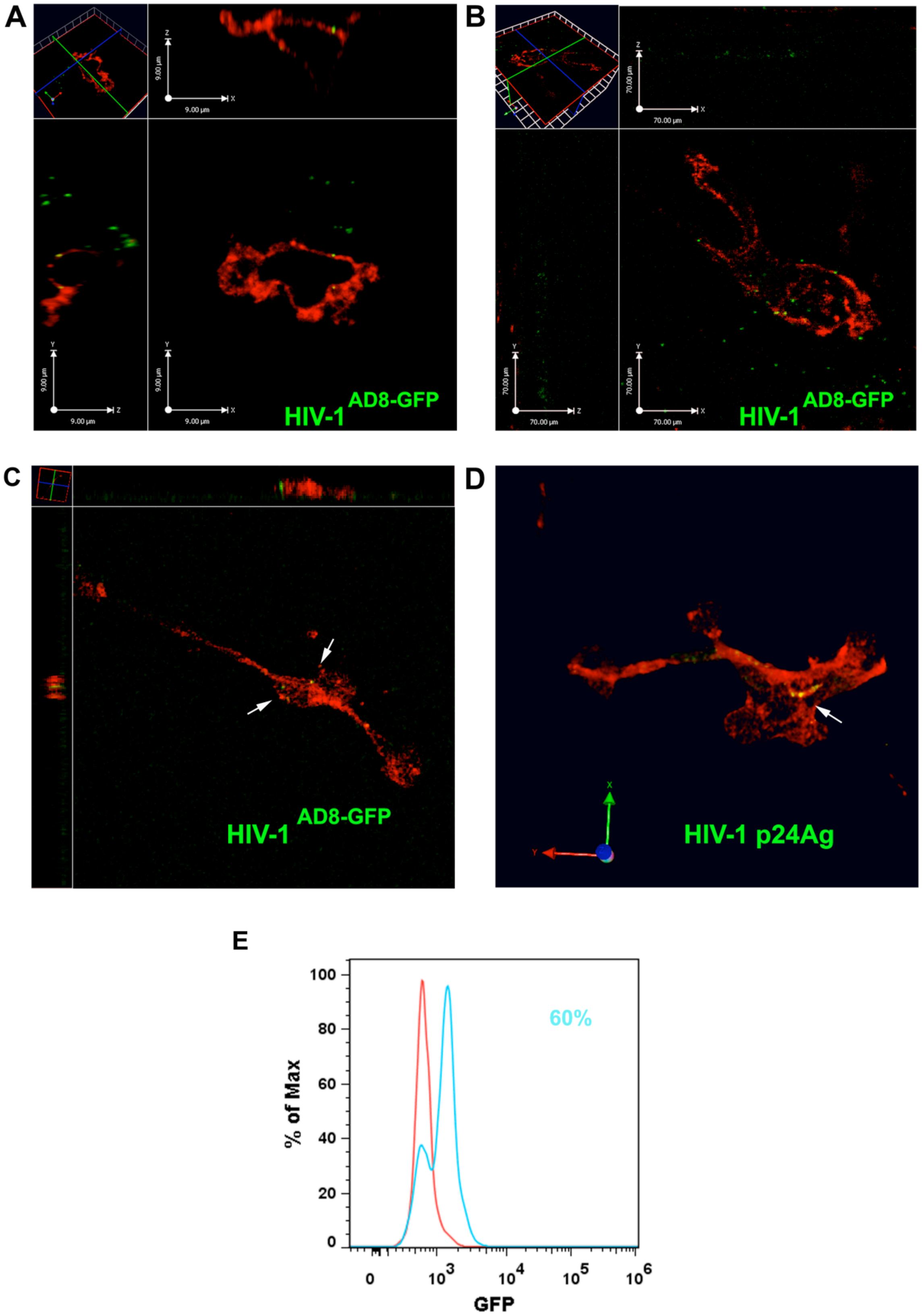
A
HIV-1 AD8-GFP
B
HIV-1 AD8-GFP
C
HIV-1 AD8-GFP
D
HIV-1 p24Ag
E
60%
% of Max
GFP
0
20
40
60
80
100
0
10^3
10^4
10^5
10^6

Fig S6 Virions are closely associated to DCs.

(A-B) R5 HIV-1$^{AD8-GFP}$ particles associated with the membrane (**A** and **B**), and inside the cytoplasm (**B**) of DCs (mouse anti-human DCSIGN-PE, red) are shown by cross sectional plane images of single DCs migrated in the Caco-2 side of the filter (Caco-2 cells not shown in the figure) after incubation with the virus (at 20 ng of p24 Ag) for 4.5 h.

(C-D) DCs (mouse anti-human DCSIGN-PE, red) detached by centrifugation from the filter of the Caco-2/DC system and settled on glass slide were bearing HIV-1 (indicated by arrows), as revealed either by GFP (**C,** cross sectional image) or by p24 (mouse anti-p24 + Alexafluor488 rabbit anti-mouse IgG) Ag labeling **(D,** two-dimensional image) Scale bar indicates the magnification.

(**E**) DCs detached by centrifugation from the Caco-2/DC filter incubated with HIV-1$^{AD8-GFP}$ were extensively washed, fixed with 2% PFA and analyzed by flow cytometry. The red line show DCs detached from medium-treated transwell (negative control) and the blue line DCs detached from HIV-1^{AD8GFP}-treated transwell.

Results are of one representative experiment out of three.

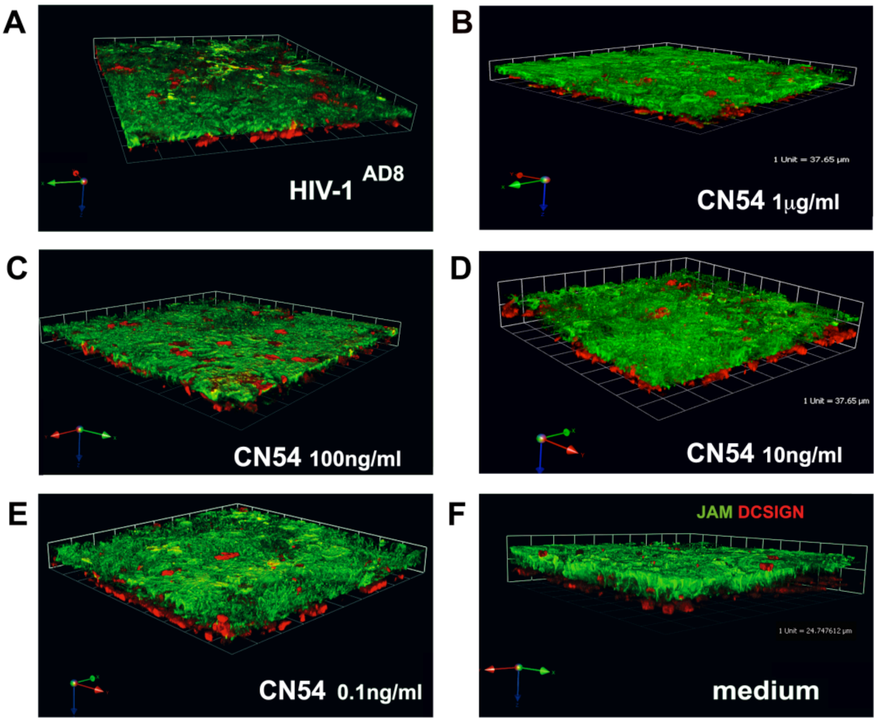

Fig S7. DCs migration across the epithelial monolayer in response to HIV-1 timeric gp140 protein is dose-dependent.

The apical side of the Caco-2/DCs co-culture system was incubated for 1.5 h either with HIV-1^{AD8} (**A**, positive control, 20 ng of p24 Ag), or with decreasing concentrations of soluble HIV-1 CN54 gp140 trimeric protein (**B**-**E**) or medium (**F**, negative control). Three-dimensional renderings from representative fields of the Caco-2/DCs culture labeled with mouse anti-human JAM + Alexafluor 488 goat anti-mouse IgG (epithelial cells; green) and mouse anti-human DC-SIGN-PE (DCs; red). Results are of one representative experiment out of three.

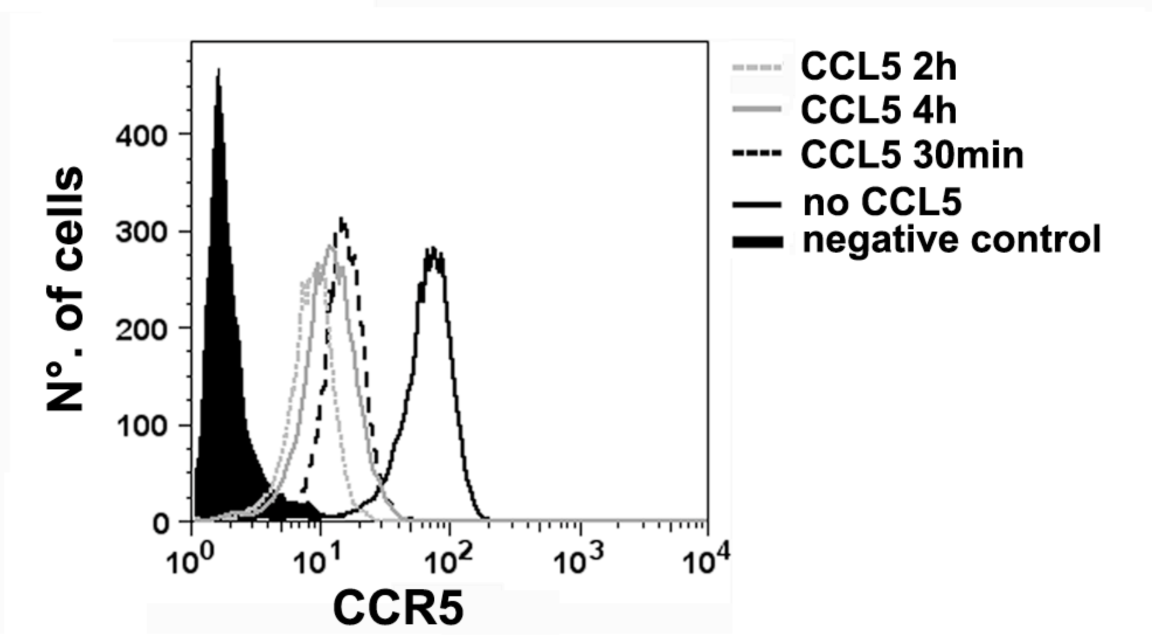

Fig S8. **Down-regulation of CCR5 receptor on DCs treated with CCL5.**

Down-regulation of CCR5 expression on DCs was observed already after 30 min with a maximum peak after 2 h of incubation with CCL5. Cytofluorimetry analysis of the surface expression level of CCR5 on DCs was performed after treatment with CCL5 (200 ng/ml) for 30 min, 2 h or 4 h or with culture medium. Cells were labeled with mouse anti-human CCR5-FITC (clone 2D7). Unstained cells were used as negative control. Results are of one representative experiment out of three.

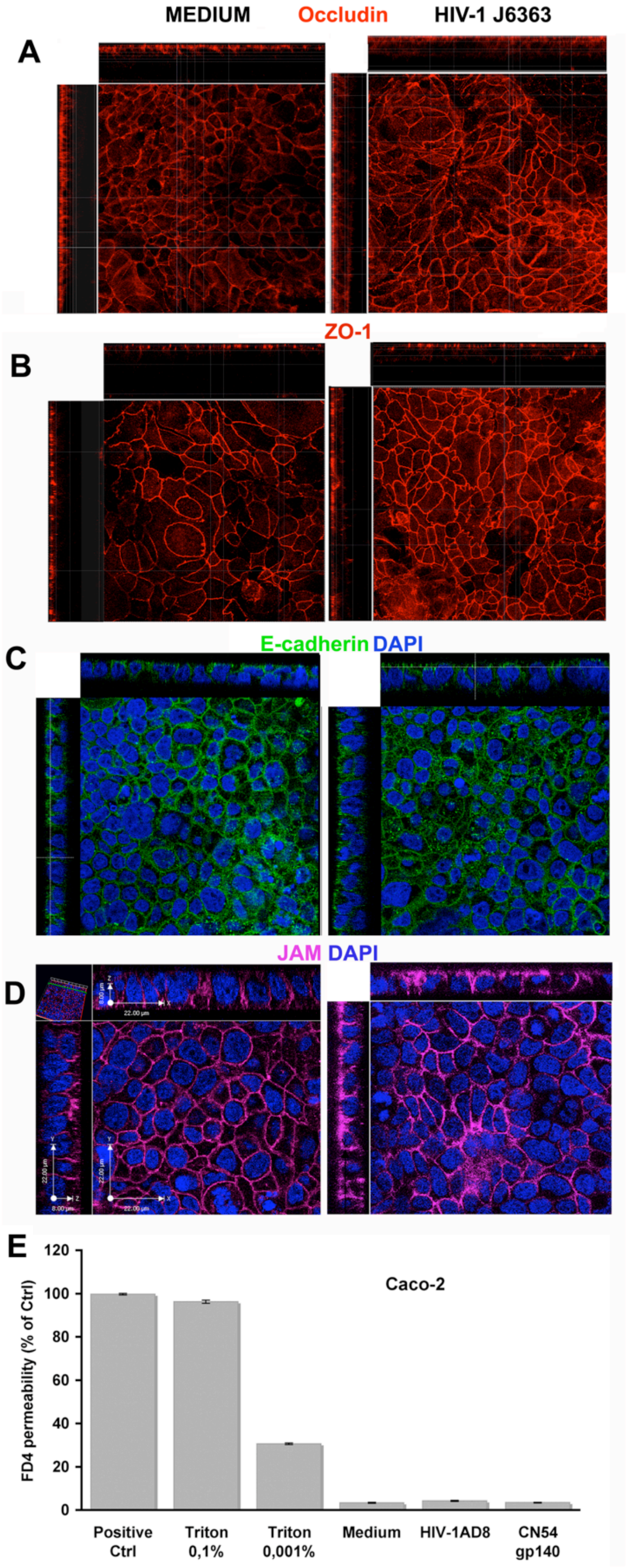
MEDIUM
Occludin
HIV-1 J6363
A
ZO-1
B
E-cadherin DAPI
C
JAM DAPI
D
E
Caco-2
FD4 permeability (% of Ctrl)
120
100
80
60
40
20
0
Positive Ctrl
Triton 0,1%
Triton 0,001%
Medium
HIV-1AD8
CN54 gp140

Fig S9 HIV-1 exposure does not alter junctional protein expression and permeability of the Caco-2 monolayer.

Confluent Caco-2 monolayer was treated with medium (left panels) or HIV-1^{J6363} (R5 strain, 4 ng of p24 Ag, right panel) for 1.5 h. Representative CM cross sectional images of single planes from z-series with staining for rabbit anti-human Occludin + Alexafluor 594 goat anti-rabbit IgG (**A**) rabbit anti-human ZO-1 + Alexafluor 594 goat anti-rabbit IgG (**B**), mouse anti-human E-Cadherin + Alexafluor 488 goat anti-mouse IgG2a (**C**), and mouse anti-human JAM + Alexafluor 488 goat anti-mouse IgG (**D**) are shown. DAPI stained the nuclei in C and D. Results are of one representative experiment out of three.

(**E**) The permeability of the monolayer to FD4 (FITC-dextran 4 kDa, at 250 μg/ml) was comparable when incubated in the presence of medium, HIV-1^{AD8} (24 ng of p24 Ag) and CN54 gp140 trimeric protein (100 ng/ml). Results are shown as percentage of the positive control (FD4 added to the filter without cells) and are 3.82% ± 0.18, 3.98 ± 0.19 and 3.67% ± 0.23 for the three different conditions. Triton X100, which caused disruption of the epithelial barrier, was included at two different concentrations as further positive control. Results are mean values from triplicates ± SD from a representative experiment out of three.

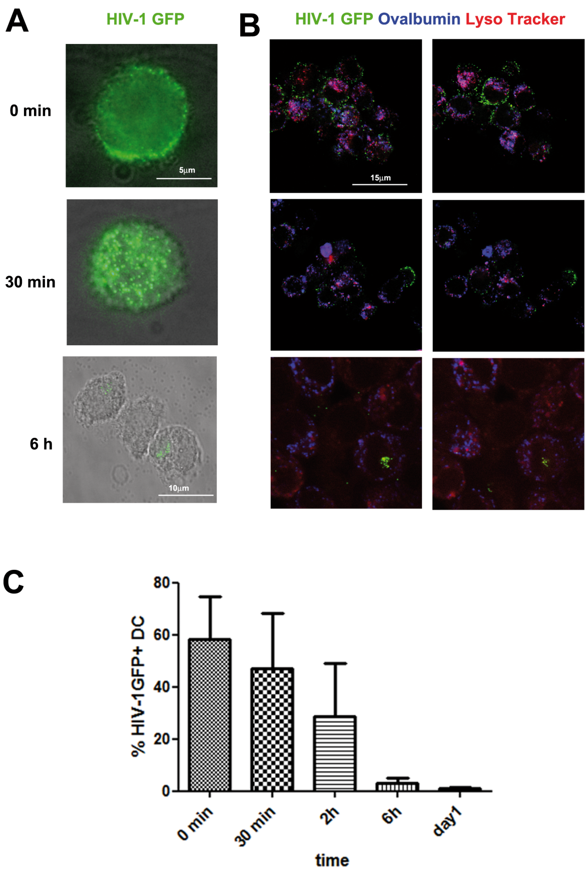

Figure S10 HIV-1 is rapidly internalized by DCs and is not redirected to an acidic lysosomal compartment.

DCs detached from transwell following 1,5 h of incubation with HIV-1^{AD8GFP} (green), in presence or absence of ovalbumin-Alexa Fluor647 (blue) and Lyso Tracker (red), were washed and fixed at the indicated time points. Note that Lyso Tracker label acidic organelles in live cells.

(**A**) Cells were analyzed by immunofluorescence microscopy. Overlapping with the bright field is shown.

(B) Shown are two representative single plane cross sectional images, taken along the z-axis of the cells analyzed at CM. Results are from one representative experiment out of three.

(**C**) DCs were analyzed by flow cytometry at the indicated time points for the expression of GFP. Mean ± SD of three different experiments is shown.

Table S1

Kinetics of HIV-1 transcytosis across Caco-2 cells.

	**Incubation time (min)**	**p24 antigen positive/total replicates (%)**	
		**HIV-1^{AD8}**	**HIV-1$^{pNL4.3}$**
**Caco-2**	30	2/9 (22)	2/9 (22)
	60	4/9 (44)	5/9 (55)
	180	9/9 (100)	9/9 (100)
	270	9/9 (100)	9/9 (100)
**Caco-2 / DCs**	30	0/9 (0)	0/9 (0)
	60	3/9 (33)	2/9 (22)
	180	9/9 (100)	8/9 (89)
	270	9/9 (100)	9/9 (100)

Footnote to Table S1

Caco-2 cells monolayer alone (Caco-2) or with DCs (Caco-2/DCs) adhered to the basal side of the filter was incubated with cell-free R5 HIV-1^{AD8} or X4 HIV-1$^{pNL4.3}$ (at 20 ng of p24 Ag) in 3 replicate cultures and in the presence of PBMCs in the basal chamber. After 30, 60, 180 and 270 min the apical chamber was removed and PBMCs left in culture for additional 7 days. HIV-1 virus production by PBMCs ranged between 0,2 ng/ml and 1,2 ng/ml of p24 Ag measured by ELISA and was determined as the difference between day 7 and day 0. Shown are the results of three experiments performed in triplicate.
